# Computational and Empirical Studies Predict *Mycobacterium tuberculosis*-Specific T Cells as a Biomarker for Infection Outcome

**DOI:** 10.1371/journal.pcbi.1004804

**Published:** 2016-04-11

**Authors:** Simeone Marino, Hannah P. Gideon, Chang Gong, Shawn Mankad, John T. McCrone, Philana Ling Lin, Jennifer J. Linderman, JoAnne L. Flynn, Denise E. Kirschner

**Affiliations:** 1 Department of Microbiology and Immunology, University of Michigan Medical School, Ann Arbor, Michigan, United States of America; 2 Department of Microbiology and Molecular Genetics, University of Pittsburgh, Pittsburgh, Pennsylvania, United States of America; 3 Robert H. Smith School of Business, University of Maryland, College Park, Maryland, United States of America; 4 Department of Pediatrics, Children’s Hospital of the University of Pittsburgh of UPMC, Pittsburgh, Pennsylvania, United States of America; 5 Department of Chemical Engineering, University of Michigan, Ann Arbor, Michigan, United States of America; Ryerson University, CANADA

## Abstract

Identifying biomarkers for tuberculosis (TB) is an ongoing challenge in developing immunological correlates of infection outcome and protection. Biomarker discovery is also necessary for aiding design and testing of new treatments and vaccines. To effectively predict biomarkers for infection progression in any disease, including TB, large amounts of experimental data are required to reach statistical power and make accurate predictions. We took a two-pronged approach using both experimental and computational modeling to address this problem. We first collected 200 blood samples over a 2- year period from 28 non-human primates (NHP) infected with a low dose of *Mycobacterium tuberculosis*. We identified T cells and the cytokines that they were producing (single and multiple) from each sample along with monkey status and infection progression data. Machine learning techniques were used to interrogate the experimental NHP datasets without identifying any potential TB biomarker. In parallel, we used our extensive novel NHP datasets to build and calibrate a multi-organ computational model that combines what is occurring at the site of infection (e.g., lung) at a single granuloma scale with blood level readouts that can be tracked in monkeys and humans. We then generated a large *in silico* repository of *in silico* granulomas coupled to lymph node and blood dynamics and developed an *in silico* tool to scale granuloma level results to a full host scale to identify what best predicts *Mycobacterium tuberculosis* (Mtb) infection outcomes. The analysis of *in silico* blood measures identifies *Mtb*-specific frequencies of effector T cell phenotypes at various time points post infection as promising indicators of infection outcome. We emphasize that pairing wetlab and computational approaches holds great promise to accelerate TB biomarker discovery.

## Author Summary

Tuberculosis (TB) is a disease that is caused by infection after inhaling the bacterium *Mycobacterium tuberculosis*. Not everyone infected with TB bacteria becomes sick. As a result, two TB-related conditions have been categorized: latent TB infection (not sick but still harboring the bacteria) and active TB disease. If not treated properly, active TB disease can be fatal. Almost 1.3 million die of TB worldwide each year, with ~8,6 million new infections in 2013. No effective vaccine is available to protect against TB and treatment of infection with multiple antibiotics is lengthy (6–9 months), with non-compliance being a major factor for the emergence of drug-resistant strains. A key step in developing effective vaccines and possibly shorter treatment regimens is the ability to identify biomarkers that correlate prognosis and progression to infection (similar to how cholesterol levels are a measure of heart health). In this study we show how pairing computer modeling, statistics and mathematics with datasets derived from non-human primate studies can accelerate biomarker discovery, and offer a new approach to identifying correlates of protection that will be useful in clinical practice, particularly in developing countries where TB is most prevalent.

## Introduction

*Mycobacterium tuberculosis* (Mtb) continues to be a global public health threat, responsible for 1.3 million deaths due to tuberculosis (TB) and 8.6 million new infections in 2013 [1]. While only 10% of infected individuals develop clinically active TB, the other 90% harbor bacteria and are considered to be clinically latent [2] (latent TB, or LTBI). Clinically latent individuals can undergo reactivation to active TB, and thus serve as a large reservoir for disease transmission. A major hurdle in controlling TB is the lack of accurate biomarkers that correlate prognosis and progression to infection [3,4,5,6].

Identification of biomarkers for both infectious and non-infectious diseases is a focus of much current biomedical research. While blood or urine can be obtained from patients to measure biomarkers, events occurring in these physiological compartments may not accurately reflect dynamics at sites of infection, such as lungs [7]. We recently showed that during Mtb infection, T cell responses in blood do not consistently reflect T cell responses within granulomas, sites of Mtb infection in lungs [8]. Biomarkers associated with the observed spectrum of infection, ranging from control of infection (LTBI) to clinically active TB [9], are unknown. This lack of understanding is present in settings of both natural and vaccine-induced immunity [6]. Here we focus our study in a natural immunity setting.

Non-specific markers of inflammation, when considered alone, do not have sufficient predictive value for clinical use in TB [3,4]. For decades, the Tuberculin Skin Test (TST) has been the most common diagnostic tool for Mtb exposure. However, lack of specificity for detection of active TB disease, inability to distinguish between BCG vaccination and Mtb infection, and inability to provide insight into disease progression limit the predictive power of TST [3,4,5]. IFN-γ Release Assays (IGRA), which measure Mtb-specific release of IFN-γ from blood cells, have higher specificity for detection an ongoing TB infection (~80%) [10] but fail both as a useful correlate of vaccine-induced protection and a reliable predictor of disease progression (i.e., due to low sensitivity or true positive rate) [3,4]. Recent association studies suggest that ratios of various T cell subpopulations in blood (e.g., CD4+ vs CD8+ T cells) may help distinguish among stages of TB progression [2,11,12,13]. Time course data in humans are typically only available from blood, and not from sites of Mtb infection (lungs or lymph nodes (LNs). In addition, it is challenging to determine the time of exposure and infection progression status in humans, limiting the ability to use these samples for predictive studies.

Gene expression profiling [2,14,15], microRNA and metabolism-based discovery [16,17], and plasma antibody profiling in Mtb infected humans [18] and animal models [3,19] have been paired with data mining and machine learning tools to uncover potential TB biomarkers. Imaging has been recently used successfully as a diagnostic and prognostic tool in TB studies (i.e., PET/CT scan), both in the context of natural infection [20,21] and drug-treatment [22,23]. While informative, these studies have thus far been unable to determine either a single or suite of practical biomarkers, or predictive correlates of protection that would be useful in clinical practice, particularly in developing countries where TB is most prevalent. This is likely due to the complexity of TB disease, that blood may not reflect lung infection dynamics [8], intrinsic limitations in *ex vivo* studies and limited availability of longitudinal *in vivo* data. Moreover, a spectrum of infection outcomes overlying binary classifications of active TB and latent infection has been identified, making biomarker discovery for infection status even more challenging [9].

**Fig 1:**
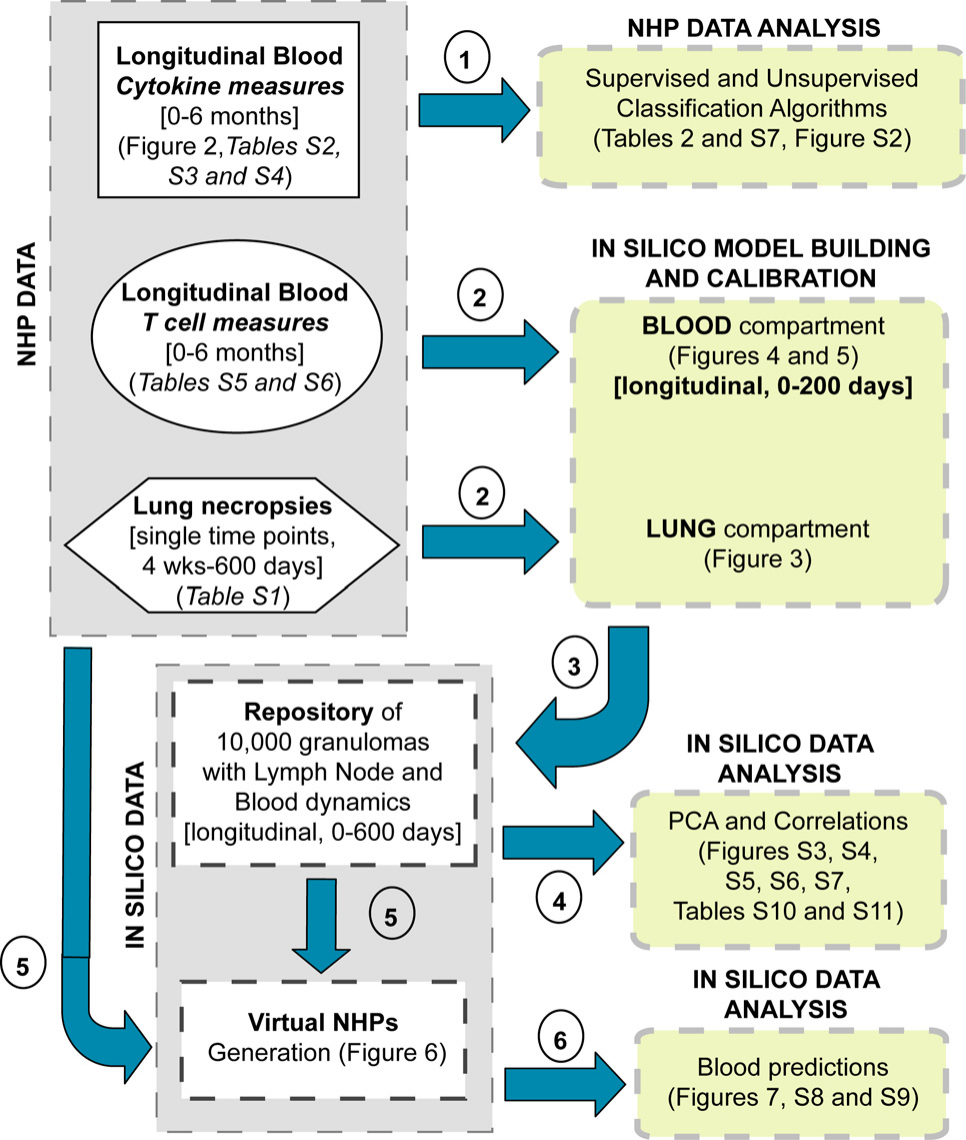
Methodology roadmap. The left side of the diagram (gray boxes) summarizes all of the datasets generated in this study. We have generated experimental datasets from blood samples and lung necropsies of non-human primates (NHPs), and datasets generated by computational model simulations (*in silico* data). The right side of the diagram (yellow boxes) represents the analyses performed on each dataset. Each dataset is displayed by a different shape. The blue arrows point to the type of analysis performed on each dataset. The circled numbers represent the chronological order of operations (referred to as steps in the text). Details on which Fig or table in the manuscript contain a dataset or analysis are given in each box.

Here we present an integrated experimental and computational modeling approach toward the discovery of TB biomarkers with the goal of predicting Mtb infection outcomes. Fig 1 shows a methodology roadmap of the different datasets generated and analyses performed in this study. First, using machine learning, immunologic data from blood of Mtb-infected cynomolgus macaques was interrogated for biomarkers that would predict infection outcome. The analysis of the NHP blood datasets did not identify any potential TB biomarker. A separate dataset was then used to help build and calibrate a computational model of the immune response during Mtb infection. Our unique multi-scale and multi-physiological compartmental computational model generates *in silico* data on dynamics of infection in both blood and lung, capturing formation of independent granulomas in lungs and at the same time T cell profiles in blood. We then constructed virtual non-human primate hosts based on granuloma data and infection status from macaques, and used these models to identify potential biomarkers that can predict infection outcome. We found that Mtb-specific frequencies of effector T cell phenotypes (i.e., both CD4+ and CD8+) in the blood can be targeted to distinguish infection outcomes, with a clear separation between median trajectories of active versus latent TB late during infection progression (~300 days).

## Results

To help the readers navigate through the many datasets generated and the different analyses performed in this study, we provide a detailed methodology roadmap (Fig 1). Each step labeled in Fig 1 refers to a section in the Results, with emphasis on the order in which they have been performed.

### A range of classification algorithms fails to reveal markers of disease outcome in a novel non-human primate dataset

**Fig 2:**
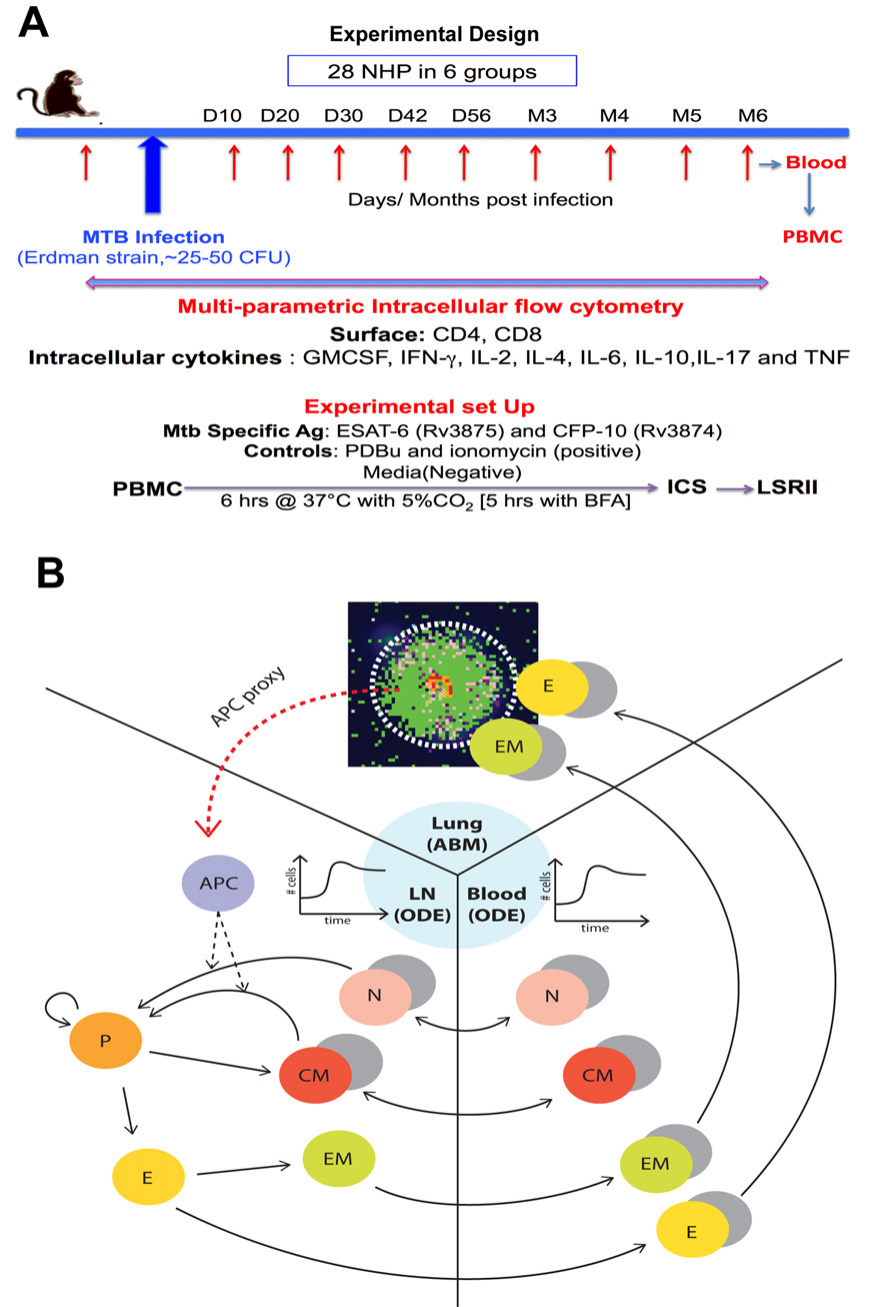
Experimental design and computational model. **(A)** Experimental design for data collection and measurement on the 28 Non-Human Primate. All the datasets are available online as Supporting Information (S2–S6 Tables). **(B)** Schematic representing the three compartments captured by our computational model. The emphasis is on describing all the lymphocyte phenotypes (both CD4+ and CD8+) tracked during the analysis. The cells populate three different compartments/organs: lung, lymph node and blood. The lung is modeled as an Agent-Based Model (ABM), while the blood and the lymph node are modeled as an Equation-Based Model (EBM), namely as an Ordinary Differential Equation (ODE) system. For most of the phenotypes, both Mtb-specific (colored) and non Mtb-specific (grey) cells are tracked. APC: represented as a proxy in the computational model (see Materials and Methods section and S1 and S2 Texts for details).

We assessed levels of CD4+ and CD8+ T cells (and memory subsets based on the expression of the two markers CD45RA and CD27, see Table 1 for details) and their cytokine production in the blood of 28 non-human primates (NHPs) (*cynomologus macaques*) at 10 time points over the course of experimental Mtb infection (up to 6 months post infection) (Fig 2A, Table 1, S1–S6 Tables). Such an extensive time course of sampling in the NHP model of TB, which recapitulates human Mtb infection, has not been previously available for biomarker studies. These NHPs were clinically classified as having either latent infection or active TB disease as previously described [24,25], but exhibit a range of outcomes encompassing these clinical classifications [26]. S1 Fig shows representative flow cytometry plots outlining gating strategies employed in assessing T cell levels.

**Table 1:** Summary of the NHP experimental machine learning, computational model calibration and scaling-to-host predictions.

Dataset	Supplementary Tables	# of animals (NHPs)	Days sampled	Data Collected	Data Used For
**NHP classification+CFU/gran data (Lung)**	**S1 Table**	43(20 Active TB, 23 Latent TB)	One time point at necropsy	• NHP ID • Outcome classification • Time of necropsy (days) • Number of granulomas • CFU/gran data	• Model Calibration (lung) • Scaling to host
**Single Cytokine dataset (Blood)**	**S2 Table**	28(14 Active TB, 14 Latent TB)	0, 10, 20, 30, 42, 56, 90, 120, 150 and180	**Single Cytokines on CD4+ and CD8+ T cells levels** 1. CD4 markers (8): GMCSF , IFNγ , IL-2 , IL-10 , TNF, IL-17, IL-6, IL-4 2. CD8 markers (9): CD-107 , GMCSF , IFNγ , IL-2 , IL-10 , TNF, IL-17, IL-6, IL-4	• Supervised and Unsupervised Classification • Features =8x10+9x10=170
**Multiple Cytokine dataset (Blood)**	**S3 Table**	19(10 Active TB, 9 Latent TB)	10, 20, 30, 42, 56, 90, 120, 150 and 180	**Multiple Cytokines on CD4+ and CD8+ T cells levels** 1. CD4 markers (5): GMCSF , IFNγ , IL-2 , IL-10 , TNF 2. CD8 markers (6): CD-107 , GMCSF , IFNγ , IL-2 , IL10 , TNF	• Supervised and Unsupervised Classification • Features = 5x9+6x9=99
**Memory Single Cytokines dataset (Blood)**	**S4 Table**	28(14 Active TB, 14 Latent TB)	0, 10, 20, 30, 42, 56, 90, 120, 150 and 180	**Single Cytokines on CD4+ and CD8+ T cells phenotypes** (Naïve-N [CD45RA+ CD27+], Effector-E or Terminally Differentiated-TD [CD45RA+CD27-], Central Memory-CM [CD45RA-CD27+] and Effector Memory-EM [CD45RA-CD27-]) 1. CD4 markers (8): GMCSF , IFNγ , IL-2 , IL-10 , TNF, IL-17, IL-6, IL-4 2. CD8 markers (9): CD-107 , GMCSF , IFNγ , IL-2 , IL-10 , TNF, IL-17, IL-6, IL-4	• Supervised and Unsupervised Classification • Features = 8x10+9x10=170
**T Cell dataset (Blood)**	**S5 Table**	9	0 10, 20, 30, 42, 56, 90, 140, and 167	**CD4+ and CD8+ T cell phenotypes** Naïve-N [CD45RA+ CD27+], Effector-E or Terminally Differentiated-TD [CD45RA+CD27-], Central Memory-CM [CD45RA-CD27+] and Effector Memory-EM [CD45RA-CD27-]	• Model Calibration (blood)
**ESAT-6 + CFP-10 or ESAT-6 & CFP-10 Memory T cell dataset (Blood)**	**S6 Table**	28	0, 10, 20, 30, 42, 56, 90, 120, 150 and 180	**All CD4+ and CD8+ T cell phenotypes producing any CK in response to stimulation to ESAT6 or CFP10** Naïve-N [CD45RA+ CD27+], Effector-E or Terminally Differentiated-TD [CD45RA+CD27-], Central Memory-CM [CD45RA-CD27+] and Effector Memory-EM [CD45RA-CD27-]	• Model Validation (blood)

To interrogate these unique and extensive NHP infection datasets for biomarkers (step 1 in Fig 1), we applied four supervised classification algorithms: classical and penalized discriminant analysis (LDA and PLDA), quadratic discriminant analysis (QDA) and logistic regression (full details are given in the Methods and Supplemental materials). Sensitivity, specificity and misclassification error rates (MER) are shown in Table 2 for both training and test sets. Results of the training dataset show high accuracy, even if overfitting is likely driving it (since we have 28 samples for more than 150 features). However, similar results were not observed in the test data set, where none of the four methods used was able to discriminate disease outcomes (MERs for the test set ~50%, Table 2; S2 Fig). The stark differences in predictive accuracy between the training and test datasets indicate over-fitting, which could potentially be mitigated by increasing sample size.

**Table 2:** Supervised classification algorithms results. Sensitivity, Specificity and Misclassification Error Rates are shown for training and test sets. 1000 repeated trials have been performed (as described in the Methods) for each classification algorithm. (A) results for the *single cytokine dataset*. (B): results for the *memory cytokine dataset*. (C): results for the *multiple cytokine dataset*.

(A) Single Cytokines Dataset (see S2 Table)				(B) Multiple Cytokines Dataset (see S3 Table)				(C) Memory Phenotypes Dataset (see S4 Table)			
**LDA**				**LDA**				**LDA**			
Training Test	Sensitivity 0.73 0.56	Specificity 0.57 0.31	Misclassification Error Rate 0.35 0.49	Training Test	Sensitivity 0.73 0.59	Specificity 0.57 0.33	Misclassification Error Rate 0.35 0.49	Training Test	Sensitivity 0.83 0.33	Specificity 0.90 0.56	Misclassification Error Rate 0.13 0.50
**LDA + Variable Selection (PLDA)**				**LDA + Variable Selection (PLDA)**				**LDA + Variable Selection (PLDA)**			
Training Test	Sensitivity 0.73 0.56	Specificity 0.54 0.31	Misclassification Error Rate 0.36 0.48	Training Test	Sensitivity 0.75 0.57	Specificity 0.53 0.33	Misclassification Error Rate 0.36 0.48	Training Test	Sensitivity 1 0.03	Specificity 0.99 0.99	Misclassification Error Rate 0.45 0.50
**Logistic regression + Variable Selection**				**LDA + Variable Selection (PLDA)**				**Logistic regression + Variable Selection**			
Training Test	Sensitivity 0.73 0.47	Specificity 0.62 0.38	Misclassification Error Rate 0.36 0.49	Training Test	Sensitivity 0.75 0.57	Specificity 0.59 0.38	Misclassification Error Rate 0.35 0.48	Training Test	Sensitivity 1 0.39	Specificity 1 0.54	Misclassification Error Rate 0 0.49
**QDA**				**QDA**				**QDA**			
Training Test	Sensitivity 0.90 0.59	Specificity 0.56 0.34	Misclassification Error Rate 0.27 0.49	Training Test	Sensitivity 0.84 0.57	Specificity 0.64 0.40	Misclassification Error Rate 0.26 0.49	Training Test	Sensitivity 0.79 0.54	Specificity 0.51 0.39	Misclassification Error Rate 0.36 0.49

We next re-analyzed the blood datasets using unsupervised clustering algorithms (multidimensional scaling, Ward’s method and other hierarchical methods, see Materials and Methods for full details). These algorithms suggested that the 28 macaques can be subdivided into 3–25 optimal clusters (S7 Table), but there was no agreement among the methods in terms of what the clustering should be. The clusters contain mixtures of NHPs with active disease and latent infection, with no clear separation between subjects and no distinct delineator of infection stage. This is likely due to the overlapping spectrum of pathology present in animals that are clinically classified as having active disease or latent infection [8,9,21,22,25], as also described for humans [27]. Overall, our multiple attempts to use longitudinal blood T cell data obtained from NHPs to predict the clinical binary classification (i.e. latent infection or active TB) were not successful.

### Computational model recapitulates blood NHP data on both T cell levels and their cytokine production

Our NHP blood dataset may not be predictive of infection outcomes if blood does not reflect lung infection dynamics (as suggested by our previous work [8]) and/or if the sample size does not have the necessary statistical power to discriminate binary outcomes. In addition, TB exists on a spectrum and identifying binary outcomes may be an artificially imposed constraint and not practical [9]. These shortfalls could be mitigated using a computational model that describes the immune response to Mtb infection in three physiological compartments capturing blood, LNs and lung dynamics, calibrated with our NHP datasets (step 2 in Fig 1 and Fig 2B). Building on previous work from our group, we developed a multiscale and multicompartment model by linking our computational model *GranSim* that captures individual granuloma formation and function in the lungs using an agent-based model framework [28,29,30,31,32,33] with 2 additional non-linear ODE models capturing dynamics in both LN and blood [28,29] (Fig 2, S1 Text).

**Fig 3:**
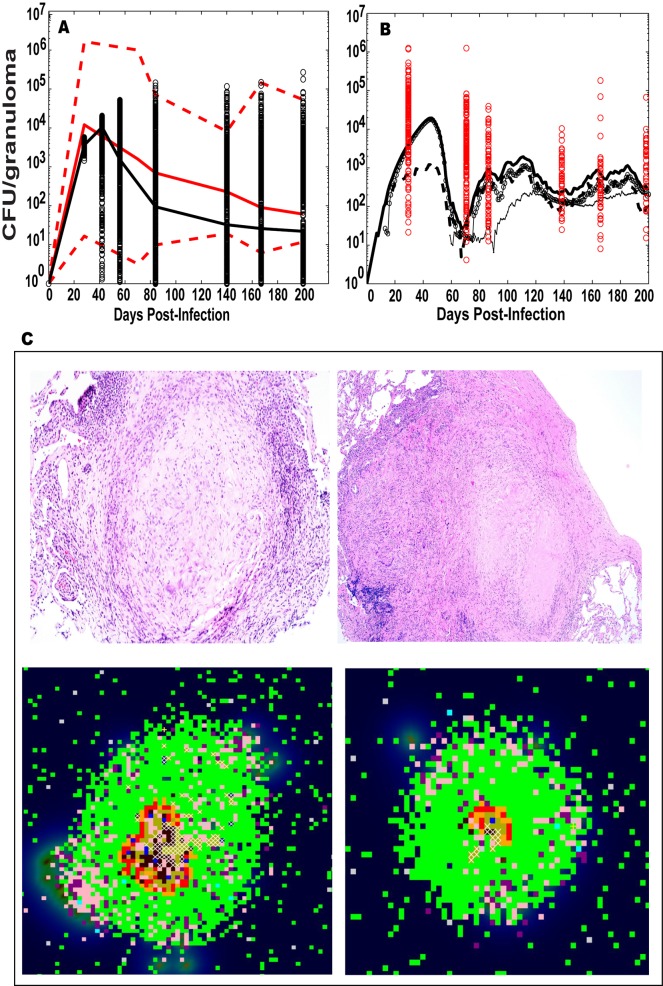
Computational model calibration: LUNG. NHP experimental data on CFU/granuloma (S1 Table) are plotted here versus the i*n silico* datasets of CFU/granuloma (lung compartment) from i*n silico* repository of 10,000 granulomas coupled to the blood and LN dynamics). Although the *in silico* dataset has time courses up to 600 days, the x-axis always shows a time span of infection up to 200 days to match the NHP blood data. The y-axis represents CFU/granuloma (A-B). (A) *In silico* dataset of time courses of CFU/granuloma generated in the lung compartment (black circles, with the black solid line representing the median trajectory) compared to experimental data on NHP CFU/granuloma (with the solid red line representing the median, and the dotted red lines representing the min and max values in the NHP data). The median trajectories for both the NHP and *in silico* data are calculated including the sterilized granulomas, while the min trajectories excluded the sterilized granulomas. (B) Mtb trajectories (total [solid thick], extracellular [solid with empty circles], intracellular [dotted] and non-replicating [solid thin]) in a representative granuloma (containment) compared to the NHP CFU/granuloma experimental data (red circles). (C) Snapshots of 4 different granulomas. The top row of Panel C is for H and E staining of two NHP granulomas. The left granuloma is from NHP 22810, CFU~40. The right granuloma is from NHP 17211, CFU~1240. Both granulomas are ~ 2mm in diameter (see S1 Table for details). The bottom row of Panel C is for *in silico* granulomas, matching lesion size and CFU/granuloma of the NHP images. Cell types displayed are the following: macrophages (resting-green, activated-blue, infected-orange, chronically infected-red), effector lymphocytes (pro-inflammatory IFN-γ producing T cells-Tγ in pink, cytotoxic T cell-Tc in purple, regulatory T cell-Treg in light blue), extracellular bacteria (olive green), vascular sources (grey and necrotic spots (white)).

**Fig 4:**
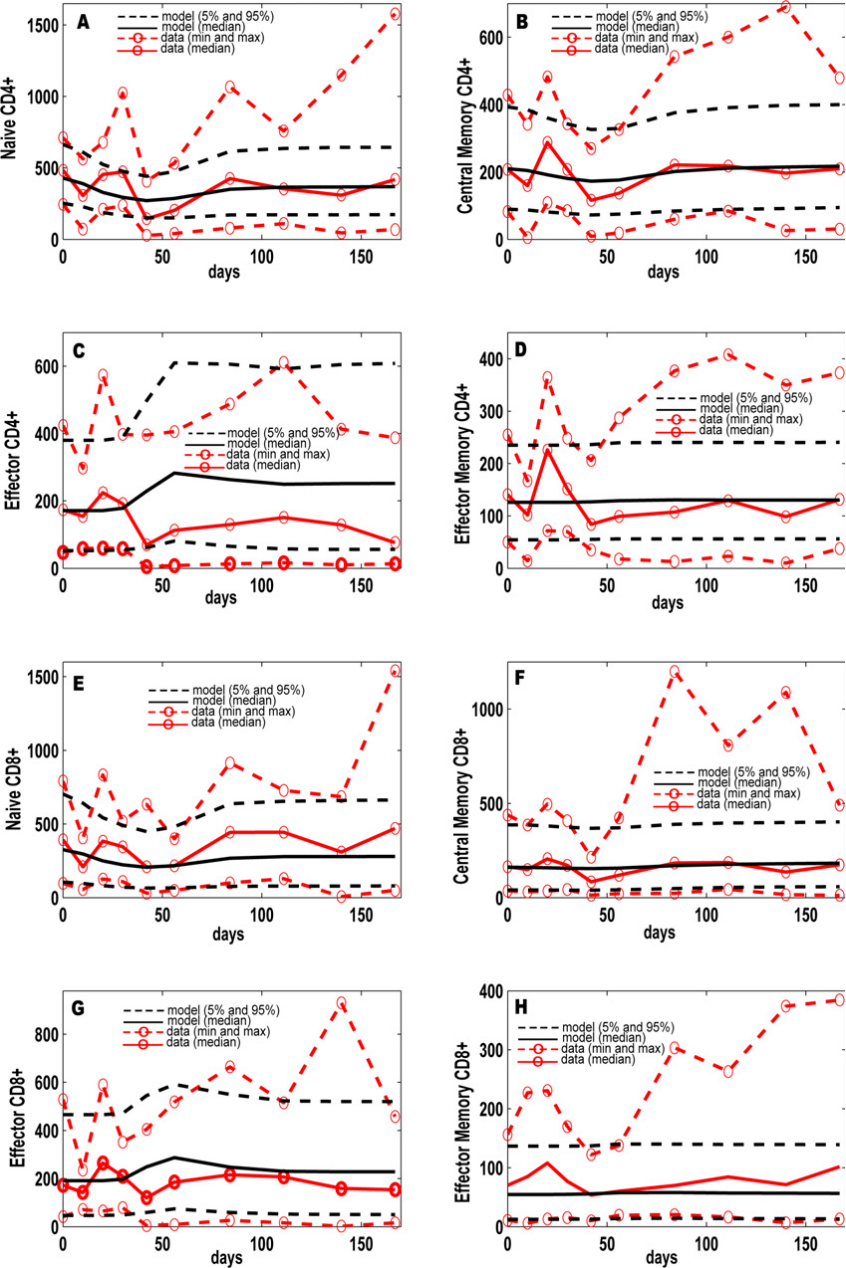
Computational model calibration to NHP data from blood. NHP experimental data on blood T cell phenotypes (S5 Table, *T cell dataset*) are plotted here versus the i*n silico* datasets of blood T cell phenotypes (blood compartment), from i*n silico* repository of 10,000 granulomas coupled to the blood and LN dynamics. Although the *in silico* dataset has time courses up to 600 days, the x-axis always shows a time span of infection up to 200 days to match the NHP blood data. The y-axis represents cells/cm^3^. (A-H) *In silico* dataset of 10,000 time courses of 8 T cell phenotypes generated in the blood compartment (black solid line [mean] and black dashed lines [5th and 95th percentiles]) compared to experimental data on T cell phenotypes in the blood of Mtb-infected NHPs (red dashed lines with red open circles, representing the min and max). For the minimum and maximum of the NHP data we chose the lowest and highest values at any time point across all the NHPs. *In silico* predictions are displayed as median (black solid line) and minimum and maximum (dashed black lines). We show Naïve CD4+ ((A) and CD8+ (E)), Central Memory CD4+ (B) and CD8+ (F)), Effector CD4+ (C) and CD8+ (G)) and Effector Memory CD4+ (D) and CD8+ (H). The *in silico* data have been obtained by summing the respective Mtb-specific and non Mtb-specific equations of the blood compartment of the computational model.

Figs 3 and 4 illustrate the calibration of our *in silico* model to our NHP dataset, with both spatial and temporal fits to distinct data from individual granulomas in lungs (Fig 3) and blood (Fig 4) (ranges for parameter values given in S8 and S9 Tables). In addition, bacterial burden per granuloma (in colony forming units, CFU) in the lung are calibrated to recently published [8,22] and unpublished NHP datasets (Fig 3A, S1 Table). Time courses for the number of Mtb bacteria from a representative contained granuloma are shown in Fig 3B. We also show a comparison between granuloma spatial distribution (e.g., location of immune cells, bacteria, necrotic center) from two NHP granuloma images and two *in silico* granuloma snapshots with similar Mtb bacteria levels and lesion size (Fig 3C). NHP T cell dynamics (S5 Table) from 9 animals were used to calibrate our *in silico* blood dynamics, and *in silico* trajectories fall within the levels of the CD4+ and CD8+ T cells from NHP blood (Fig 4).

### Computational model allows for prediction of dynamics of Mtb-specific T cells

**Fig 5:**
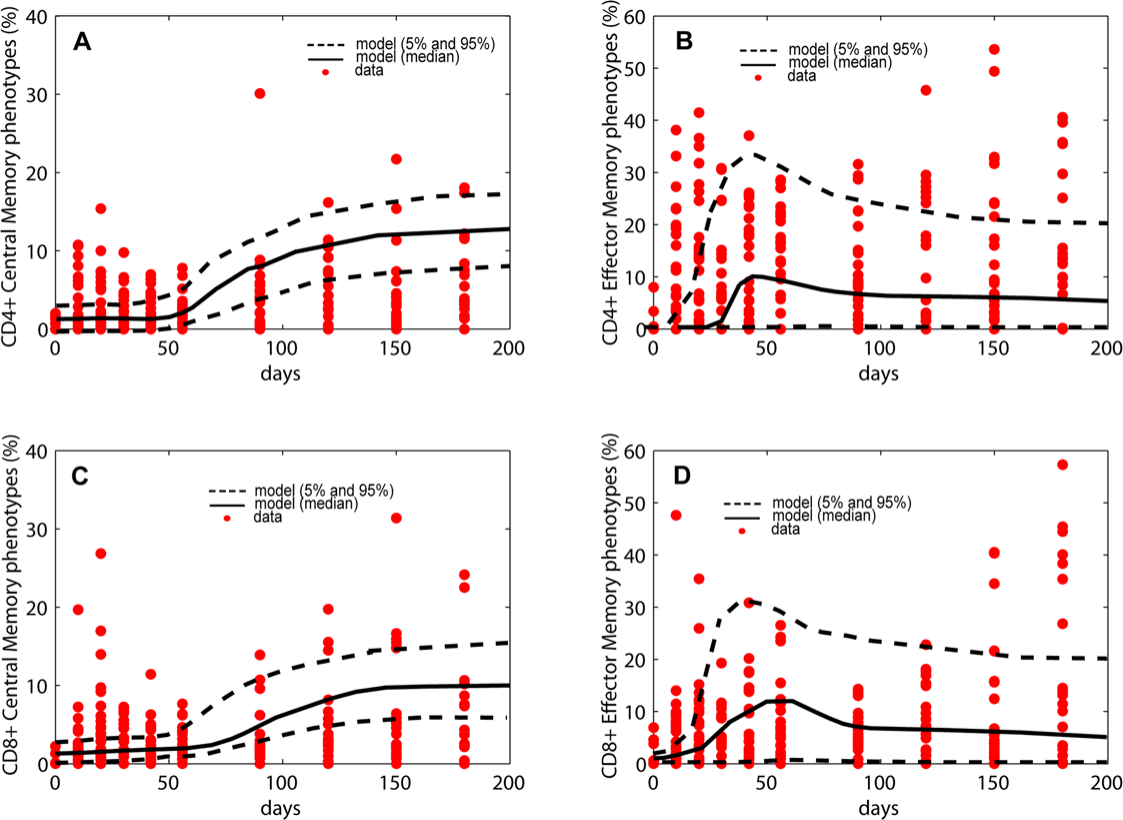
Model validation of *in silico* Mtb-specific frequencies. Trajectories over 200 days of T cell frequencies from the computational model against NHP experimental data. The *in silico* data have been generated following steps illustrated in Fig 6, as well as in the Materials and Methods section. Given the non-zero frequencies in the pre-infection stages, we used initial conditions between 0.01% and 2% for the Mtb-specific Central and Effector memory phenotypes. The red dots represent NHP experimental data, namely the frequencies of T cells producing any of the 6 cytokine measured (IFN-γ, IL-2, IL-6, IL-10, IL-17 and TNF) in response to ESAT-6 and CFP-10 stimulation (see Materials and Methods section for details and S6 Table for the data). Only 9 NHPs data are plotted here. The black solid (mean) and dashed (5th and 95th percentiles) lines represent the trajectories of the *in silico* data. *Panel A*: Frequencies of CD4+ T cell Central Memory phenotypes. *Panel B*: Frequencies of CD4+ T cell Effector Memory phenotypes (Terminally Differentiated and Effector Memory). *Panel C*: Frequencies of CD8+ T cell Central Memory phenotypes. *Panel D*: Frequencies of CD4+ T cell Effector Memory phenotypes (Terminally Differentiated and Effector Memory).

Currently, Mtb-specificity of T cells in the NHP model cannot be directly measured (for example, tetramers are not available). As a surrogate, we used our NHP blood dataset to calculate frequency of T cells that produce any of 6 cytokines measured (IFN-γ IL-2, IL-6, IL-10, IL-17 and TNF) (S6 Table) following stimulation with two Mtb-specific immunodominant antigens ESAT-6 and CFP-10 (S6 Table). These T cell frequencies are then used as proxies for the frequencies of Mtb-specific T cells (at least from the perspective of a functional response to these antigens). This does not provide a direct measure of cells that are truly Mtb-infection specific. The NHP used in this study are outbred monkeys that could have been exposed to non-tuberculous mycobacteria (NTMs, or environmental exposure) and would nonetheless respond to stimulation with these antigens prior to our infecting them with Mtb. This may explain the non-zero frequencies in the NHP dataset present in many of the memory T cell phenotypes observed early during infection (red dots, Fig 5). The computational model does not account for these potential effects.

We next used our calibrated *in silico* model to predict the spatio-temporal dynamics of Mtb-specific T cells (see Materials and Methods section for details). To parallel our computational model of virtual NHPs that would also previously be exposed to mycobacterial antigens, our simulations start with non-zero initial conditions in the same frequencies as the NHP data. Our *in silico* model predictions for frequencies of Mtb-specific Central Memory (Fig 5A and 5C) and Effector T cells (Fig 5B and 5D) are within the variation of the NHP data that derived as proxies for Mtb-specific T cell responses.

### *In silico* frequencies of Mtb-specific Effector CD4+ and CD8+ T cells in blood correlate to CFU per granuloma in the lung

We next apply data mining techniques and correlation methods on these *in silico* datasets for discovery of potential biomarkers (step 4 in Fig 1). Since our computational model can predict dynamics of Mtb-specific cells in blood, lung and LNs, we use our model to generate large *in silico* datasets (10,000 virtual granuloma), pairing lung outputs (i.e., CFU/granuloma dynamics) with blood measures (immune cell dynamics) over the time course of infection (step 3 in Fig 1). We first apply principal component analyses (PCA) to the *in silico* blood readouts only (S3 Fig), and then we extend the analysis to the 3 compartment readouts combined together (e.g., blood, lymph node and single granuloma in the lung, see S11 Table and S4 and S5 Figs). If the analysis is performed only on Mtb-specific T cell variables in the blood, the top 2 principal components alone explain ~70% of dataset variability (S3A Fig). Even if no cluster emerges (S3B Fig), the top 2 principal components are dominated by Mtb-specific effector CD4+ and effector CD8+ T cells, as early as 42 days post infection, as well as Mtb-specific Central Memory CD4+ and CD8+ T cells later during infection (S3C Fig). To explore this finding further, we narrowed the analysis of the *in silico* datasets by correlating virtual Mtb-specific frequencies of T-cells in blood to virtual CFU/granuloma at the site of infection (lungs). *In silico* frequencies of Mtb-specific Effector CD4+ and CD8+ T cells in blood predict well the granuloma-scale bacterial burden, with a clear separation between low vs high CFU/granuloma (all results shown in supplement at the granuloma scale- S6 and S7 Figs, S10 Table). However, since infection usually results in multiple granulomas within a single host, a host scale-readout, not a single granuloma-scale readout, would be more useful. We provide such an analysis below.

### *In silico* frequencies of Mtb-specific Effector CD4+ and CD8+ T cells in blood predict host infection outcomes

We next generate host-scale predictions by combining NHP lung necropsy data and the *in silico* repository described in the previous section (Fig 1, step 5). Our main assumption is that host-scale clinical outcomes can be determined by the combined effect of a host’s heterogeneous granuloma bacterial burden [21]. In fact, we previously demonstrated substantial heterogeneity and variability in NHP granulomas at the bacterial and immune cell levels, both among animals and within a single animal [8,21,22]. Total bacterial burden in NHP lungs at necropsy correlates with infection outcome (i.e. NHP with active disease have higher bacterial burden than those with latent infection) [25]. The contribution of individual (and total) granulomas in a single host to factors that can be tracked in blood is not clear, and likely is responsible for the lack of correlation of T cell responses in blood with those in granulomas or with clinical status in our previous study [8].

**Fig 6:**
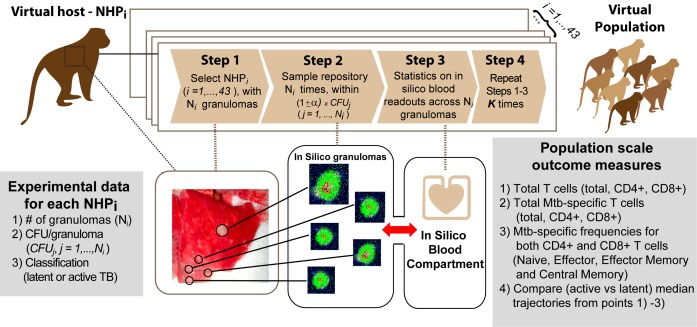
Scaling to host methodology. Experimental data on 43 Mtb-infected NHPs classified as either latent or active TB will be used to guide virtual NHP building process (see Materials and Methods section for further details). **Step 1.** One NHP out of the 43 (NHPi, i = 1, …,43) is selected and the number of granulomas (Ni) to sample from the *in silico* repository is determined, together with the CFU per each granuloma (CFUj, j = 1,….,Ni). **Step 2.** For each CFUj, we select a subset of *in silico* granulomas from the repository within the range [(1-α)xCFUj, (1+α)xCFUj]. We used α = 10%. The subset is sampled at the time point necropsy for NHPi was performed (see details on S1 Table). **Step 3.** Statistics on blood readouts are calculated (i.e., mean, median, standard deviation) and stored. **Step 4.** Steps 1–3 are repeated **K** times for the same NHPi to mimic the variability and heterogeneity in granuloma outcomes within a single host. The **K** replications are then stored and host-scale statistics are computed (i.e., mean, median, standard deviation) and combined to simulate trajectories of *in silico* blood readouts to predict infection outcomes, as shown in Fig 7.

To scale our computational model results from single lung granuloma to whole host, we first generate a large sample of virtual NHP hosts by using our *in silico* dataset to simulate NHP infections (Fig 6) that recapitulate known CFU/granuloma data (S1 Table). We generated virtual hosts that contained the number of granulomas and CFU/granuloma to match our NHP dataset (step 5 in Fig 1). For each virtual NHP host, we sample from our *in silico* repository of 10,000 granulomas (see Materials and Methods section for detail). We then combine all the sampled *in silico* granulomas and examine their corresponding *in silico* blood data on immune cell levels as generated from the computational model. This technique allows us to predict time courses of many different T cell phenotypes in the blood using our computational model (step 6 in Fig 1). Our goal here is to predict the collective behavior of latent and active TB groups rather than *in silico* blood trajectories of single NHP. Thus the scaling to host analysis does not include the computation of specificity, sensitivity and misclassification rates.

**Fig 7:**
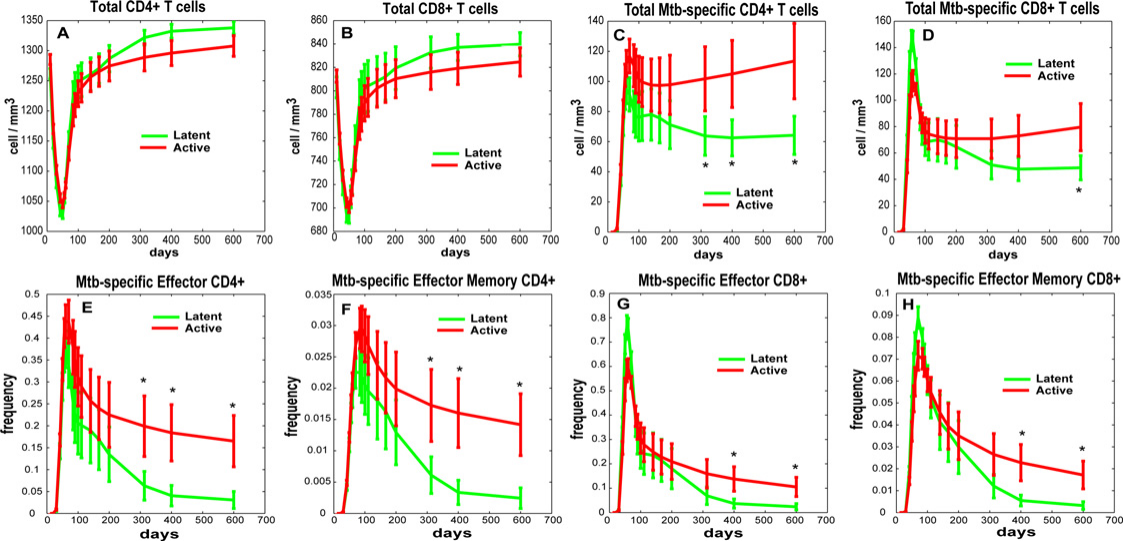
Scaling to host infection outcome predictions for Mtb-specific T cell frequencies. We built/calibrated virtual NHPs that replicate granuloma heterogeneity and variability in the lung of 43 NHPs (up to 600 days post infection, see S1 Table). Here, *in silico* blood trajectories of total T cell levels and Mtb-specific T cell frequencies grouped by clinical outcome (as shown in S1 Table) are plotted over 600 days for the same set of virtual NHPs. The *in silico* data shown here have been generated following steps illustrated in Fig 6, as well as in Materials and Methods section. The two virtual trajectories representing the 43 virtual NHPs are displayed in all the panels as mean +/- 2x(standard error). The asterisks show significant (p<0.05) student t-test between the two trajectories at the same time point. These *in silico* trajectories have been generated with zero initial conditions for the Mtb-specific T cell memory phenotypes (except for Naïve Mtb-specific T cells, see Materials and Methods section for details). No experimental data is shown here since blood measures are only available for a limited number of NHPs (I.e., 12 out o 43) and only up to 180 days post infection. *Panel A*: Total CD4+ T cell levels. *Panel B*: Total CD8+ T cell levels. *Panel C*: Total Mtb-specific CD4+ T cell levels. *Panel D*: Total Mtb-specific CD8+ T cell levels. *Panels E*: Mtb-specific frequencies of Effector CD4+ T cells. *Panels F*: Mtb-specific frequencies of Effector Memory CD4+ T cells. *Panels G*: Mtb-specific frequencies of Effector CD8+ T cells. *Panels H*: Mtb-specific frequencies of Effector Memory CD8+ T cells.

Predicted numbers of CD4+ and CD8+ T cells (including the subsets of Mtb-specific T cells) in blood were generated for 43 virtual NHP (20 latent infection, 23 active TB as in the experimental NHP dataset) (Fig 7 and S8 and S9 Figs). These predictions match the NHP blood dataset, suggesting that one cannot distinguish between active and latent TB outcomes by monitoring total T cell levels (Fig 7A–7C and S8A and S8C–S8E Fig).

In contrast, predicted Mtb-specific T cell levels in blood do allow us to distinguish infection outcomes (active vs latent) by ~300 days post infection (both CD4+ and CD8+, Fig 7C and 7D). The predicted frequencies of Mtb-specific Effector CD4+ and CD8+ T cells in virtual active vs latent hosts separate after 300 days post infection (Fig 7E and 7G). The predicted frequencies of Mtb-specific Effector Memory CD4+ and CD8+ T cells are also indicative of outcome but at later time points (Fig 7F and 7H). Overall, we predict that frequencies of Mtb-specific effector CD4+ and CD8+ T cells in blood are significantly higher (from 2- to 4- fold) in an active versus a latent Mtb-infected NHP and thus a combination of these cells and various time points post-infection should be targeted as potential biomarkers of Mtb infection progression.

## Discussion

One of the greatest tools in disease diagnosis and treatment is a robust biomarker. In TB, there been has much debate regarding whether biomarkers exist and, if so, what could serve as appropriate biomarkers [3,4,5,10,11,12,13,14,15,16,17,18,19,34,35]. To date, no biomarker (or set of biomarkers) has been shown to be useful in discriminating the extent of infection and disease in humans. One of the complications in predicting Mtb infection status is the spectrum of disease outcomes encompassed within the binary classifications of active TB and latent infection [9,36,37]. This variability in disease outcome is also paralleled by heterogeneity in granuloma outcomes both between, and within, individual NHPs.

We previously reported that a spectrum of granulomas, in terms of numbers, types, immune responses and bacterial burden are found in individual animals and among animals with active or latent infection [8,22,25]. Recent studies from our group support that progressive and healed granulomas can coexist within the same animal, with nearly all animals capable of sterilizing at least a subset of individual granulomas [21]. However, animals with active TB show a subset of lesions that do not control infection, which presumably results in dissemination [22]. Not surprisingly, given this heterogeneity within hosts, systemic T cell responses in the blood do not accurately reflect granuloma T cell responses [8].

Here we collected a unique and extensive set of phenotypic and functional T cell data in blood from 28 NHPs experimentally infected with a low dose of Mtb and with a known clinical outcome (active or latent TB). We present an approach that integrates these experimental NHP data into a computational model capturing immune dynamics in 3 physiological compartments. We apply classical data mining techniques to temporal datasets from blood that are derived from both NHP studies and from our computational model. A major benefit of using data mining techniques on computational data is that *in silico* datasets are exhaustive in both time and density, allowing for greater sample sizes and statistical power. This can rule out small sample size as a potential error in the corresponding animal study and point to other factors at play. Standard supervised and unsupervised classification algorithms returned no clear decomposition or optimal cluster distribution. This suggested that the resolution of NHP blood measures that were collected was not able to identify potential biomarkers of disease progression, possibly due to the overlap between the binary classifications of infection outcome. Moreover, if we speculate that clinical outcome of hosts can ultimately be driven by the combined activity of an individual’s granuloma burden, sampling the blood will likely reflect the average dynamics of variable local immune responses to infection. Ultimately, we were unable to identify biomarkers from NHP experimental data collected in a systemic compartment (blood), indicating the complex nature of localized lung disease in TB.

To offer complementary analyses, we built a novel and unique multi-organ computational model that tracks infection dynamics in lungs, blood and LNs, using the extensive NHP datasets distinctly for model building, calibration and validation. This allows generation of a large (on the order of thousands) parallel *in silico* dataset to mine for potential biomarkers. One aspect of our *in silico* system is the ability to accurately simulate Mtb-specific T cell frequencies over time. These data indicate that Mtb-specific effector CD4+ and CD8+ T cell frequencies in the blood could serve as potential biomarkers to predict infection outcome. Although full ranges of Mtb-specific T cell frequencies are not currently measurable in primates, many studies are now available on Mtb protein epitopes (e.g., ESAT-6, Ag85B, 16kDa, 19kDa, Hsp65, Rv1490) for the most common human HLAs (reviewed in [38]). For each epitope, ranges for frequencies of antigenic-specific T cells span from 0.05% up to 1–2%. If we assume that an entire Mtb-specific frequency repertoire can be represented by the sum of the responses to many of these epitopes, our new scaling-to-host methodology offers predictions that are quantitatively comparable to these ranges (Fig 7 and S8 Fig).

Because of the variability in human HLA molecules and the large number of potential Mtb antigens, distinguishing total Mtb-specific T cell frequencies in blood is not yet feasible, especially for CD8+ T cells. However, recent studies have identified novel epitope sequences from Mtb that may soon be targets to discriminate and quantify Mtb-specific T cell responses [38,39]. This will allow prediction of infection status or treatment outcome using peripheral blood samples from infected hosts [40,41,42,43,44,45]. For example, a recent study by Sette et al [39] provides an extensive list of Mtb-derived epitopes recognized by CD4+ T cells from healthy and LTBI individuals. The authors emphasize how CD4+ T cells from both groups recognize non-tuberculous mycobacteria (NTMs, or environmental exposure) epitopes (likely from previous exposure). This might also explain the large variability identified in the NHP T cell data derived herein (Fig 5). The list of peptides that reflect true Mtb-specific versus environmental responses is by no means complete, but we can reasonably speculate that more Mtb-specific epitopes will be identified in the near future. By measuring these pathogen-specific responses, we will have a more adequate estimate of Mtb-specific immunity generated upon infection to correlate with protection or outcomes. Moreover, by including these combinations of Mtb-specific epitopes into our computational model, we could eventually predict infection outcome earlier or discriminate among a spectrum of infection outcomes.

A recent study on MDR-TB patients shows how our approach can be viable in a clinical setting. Riou et al [40] identified a subset of activated and proliferating Mtb-specific CD4+ T cells (i.e., Ki67+ HLA-DR+) as a potential marker in peripheral blood that predicts the time to sputum culture conversion in TB patients at the start of treatment. Another recent prospective proof-of-concept study uses a novel T-cell activation marker-tuberculosis (i.e., TAM-TB) assay on cryopreserved peripheral blood mononuclear cell samples to diagnose active TB in children based on ratios of CD27 phenotype of CD4 T cells producing IFNγ in response to Mtb antigens (i.e., ESAT6 and CFP10) [42].

It has been also suggested that a shift of Mtb-specific Central Memory to Mtb-specific Effector Memory CD4+ T cells precedes the clinical diagnosis of active TB by many months [44]. If we restrict our analysis to ratios of Mtb-specific Effector Memory to Mtb-specific Central Memory T cell frequencies (*in silico* data), no significant difference can be shown between latent and active TB infection groups (see S9A Fig). However, our *in silico* predictions for the time courses of the ratios of Mtb-specific Effector to Mtb-specific Central Memory CD4+ T cells, and higher ratios in the active TB group (statistically significant only after day 300 post infection) confirm the finding of Schuetz et al (S9B Fig and [44]).

In complex diseases such as heart disease and cancer, a suite of biomarkers best predicts disease outcomes and treatment intervention points. Our studies support that for TB, a complex lifelong infection that is compartmentalized primarily in the lung, single biomarkers in blood may not be a feasible goal. Coupled to the idea that infection outcome in hosts likely occurs over a spectrum, identification of a single biomarker may be a misguided goal. Our novel systems biology approach combined with the ongoing progress to elucidate and winnow down Mtb-specific epitopes can significantly influence hypothesis generation for biomarker prediction for TB. Any *in silico* prediction can then be validated on human clinical data and animal model studies, adding important knowledge to human immunity to TB and TB intervention studies.

## Materials and Methods

### Study design

The goal of this study was to develop a methodology to identify biomarkers for TB infection outcome. Our systems biology approach integrates non-human primate (NHP) datasets together with computational modeling, allowing the generation of virtual hosts that we use for biomarker discovery. The next sections illustrate: i) the experimental datasets, ii) classification algorithms, iii) computational modeling framework, iv) *in silico* datasets analysis, v) model calibration and vi) virtual hosts generation. Refer to Fig 1 for a general roadmap of all the different datasets that have been generated and all the many analysis that have been performed in this study.

### Ethics statement

All experimental manipulations, protocols, and care of the animals were approved by the University of Pittsburgh School of Medicine Institutional Animal Care and Use Committee (IACUC). The protocol assurance number for our IACUC is A3187-01. Our specific protocol approval numbers for this project are 11090030 and 12060181. The IACUC adheres to national guidelines established in the Animal Welfare Act (7 U.S.C. Sections 2131–2159) and the Guide for the Care and Use of Laboratory Animals (8^th^ Edition) as mandated by the U.S. Public Health Service Policy.

All macaques used in this study were housed at the University of Pittsburgh in rooms with autonomously controlled temperature, humidity, and lighting. Animals were singly housed in caging at least 2 square meters that allowed visual and tactile contact with neighboring conspecifics. The macaques were fed twice daily with biscuits formulated for non human primates, supplemented at least 4 days/week with large pieces of fresh fruits or vegetables. Animals had access to water *ad libitem*. Because our macaques were singly housed due to the infectious nature of these studies, an enhanced enrichment plan was designed and overseen by our nonhuman primate enrichment specialist. This plan has three components. First, species-specific behaviors are encouraged. All animals have access to toys and other manipulata, some of which will be filled with food treats (e.g. frozen fruit, peanut butter, etc.). These are rotated on a regular basis. Puzzle feeders foraging boards, and cardboard tubes containing small food items also are placed in the cage to stimulate foraging behaviors. Adjustable mirrors accessible to the animals stimulate interaction between animals. Second, routine interaction between humans and macaques are encouraged. These interactions occur daily and consist mainly of small food objects offered as enrichment and adhere to established safety protocols. Animal caretakers are encouraged to interact with the animals (by talking or with facial expressions) while performing tasks in the housing area. Routine procedures (e.g. feeding, cage cleaning, etc) are done on a strict schedule to allow the animals to acclimate to a routine daily schedule. Third, all macaques are provided with a variety of visual and auditory stimulation. Housing areas contain either radios or TV/video equipment that play cartoons or other formats designed for children for at least 3 hours each day. The videos and radios are rotated between animal rooms so that the same enrichment is not played repetitively for the same group of animals.

All animals are checked at least twice daily to assess appetite, attitude, activity level, hydration status, etc. Following *M*. *tuberculosis* infection, the animals are monitored closely for evidence of disease (e.g., anorexia, weight loss, tachypnea, dyspnea, coughing). Physical exams, including weights, are performed on a regular basis. Animals are sedated prior to all veterinary procedures (e.g. blood draws, etc.) using ketamine or other approved drugs. Regular PET/CT imaging is conducted on most of our macaques following infection and has proved very useful for monitoring disease progression. Our veterinary technicians monitor animals especially closely for any signs of pain or distress. If any are noted, appropriate supportive care (e.g. dietary supplementation, rehydration) and clinical treatments (analgesics) are given. Any animal considered to have advanced disease or intractable pain or distress from any cause is sedated with ketamine and then humanely euthanatized using sodium pentobarbital.

### Experimental datasets

Twenty-eight cynomolgus macaques (*Macacca fasicularis*) were infected with a low dose of Mtb (Erdman strain, ~25–50 CFU) and monitored clinically for signs and symptoms of TB up to six months post infection. Infection was confirmed by tuberculin skin test conversion and/or lymphocyte proliferation assay six weeks post-infection. The macaques were classified (as previously described in [22,23,25]) to have either clinically latent TB infection or active TB (S1 Table, third column) based on clinical, radiological and microbiologic criteria. Peripheral blood was collected at time-points: pre Mtb infection (only for 9 NHPs) and at days 10, 20, 30, 42, 56, 90 (or M3, 3 months), 120 (or M4), 150 (or M5) and 180 (or M6) post infection. PBMC were isolated, stimulated with peptide pools of Mtb specific antigens ESAT-6 and CFP-10 (10μg/ml of each peptide) or phorbol dibutyrate (PDBu) and ionomycin as positive control and media as negative control, in the presence of Brefaldin A (BD biosciences) for 6 hours at 37°C with 5%CO_2_. Multi-parametric intracellular flow cytometry was performed on fresh PBMC to assess CD4 and CD8 T cell cytokine profiles (GMCSF (clone: M5D12), IFN-γ (B27), IL-2(MQ1-17H12), IL-4 (8D4-8) IL-6 (MQ2-6A3), IL-10 (JES3-9D7), IL-17(64CAP17) and TNF (MA611)) during the course of Mtb infection. S1 Fig shows representative flow cytometry plots where we outline our gating strategies employed in the analysis of T cells in PBMC.

We generated four different datasets: a *single cytokine* dataset (where each cell was labeled for a single cytokine, S2 Table), a *multiple cytokine* dataset (where each cell was labeled for the simultaneous presence of more than 1 cytokine, S3 Table), a *memory single cytokine dataset* (S4 Table, where the same cytokines profile of the *single cytokine* dataset is stratified by CD4+ and CD8+ memory sub-populations based on the expression of CD45RA and CD27, namely Naïve-N [CD45RA+ CD27+], Central Memory-CM [CD45RA-CD27+], Effector Memory-EM [CD45RA-CD27-] and Terminally Differentiated-TD or Effector-E [CD45RA+CD27-] and a *T cell dataset* (where only CD4+ and CD8+ memory phenotypes levels are measured, S5 Table). S6 Table summarizes the frequencies of ESAT6 or CFP10-stimulated memory T cells that produced any cytokine (i.e., IFN-γ, IL-2, IL-6, IL-10, IL-17 and TNF) upon stimulation.

All these datasets are displayed in Fig 1 under the grey box labeled NHP data, with a detailed description given in Table 1. The complete datasets are available as supplementary material (S1–S6 Tables). The experimental design is described in Fig 2A. The time points measured in the *T cell dataset* (S5 Table) differ slightly from the other three datasets (S2–S4 Tables, see Table 1 for details on the four datasets). The first three datasets were used for machine learning (step 1 in Fig 1), while the T cell dataset (S5 Table) was used to build (Fig 2B) and calibrate the *in silico* model for the blood compartment of the computational model ((step 2 in Fig 1 and Fig 4). Moreover, the ESAT6-CFP10 Memory T cell dataset (S6 Table) was used for a tentative model validation of the Mtb-specific frequency predictions of our *in silico* model (Fig 5). S1 Table describes on all the NHPs enrolled in the study. A total of 58 NHPs have been monitored (all have been classified as either latent [green] or active [red] TB), 28 of which have immunologic data from blood (*single*, *multiple and memory cytokine datasets*, as well as *T cell dataset*). As for the number of granulomas and CFU per granuloma data, S1 Table has all the details on the 43 NHPs that have been necropsied (with time of necropsy included), 12 of which are included in the 28 NHPs of the blood data. S1 Table was used to calibrate the lung compartment of the computational model (step 2 in Fig 1 and Fig 3).

### Data preparation for data mining

The data for some cytokine profiles was imputed (ascribed) using the techniques described in [46], which relies on a “nearest neighbor” or local averaging approach to fill in missing values. The imputation approach is stochastic in nature [46], and hence is sensitive to an initial random seed. The results presented below were obtained by averaging over 1000 trials of imputing and classifying. The results were qualitatively unchanged regardless of whether cytokine profiles were scaled to have unit variance. The analysis was performed in R (version R 2.14.2) and in Matlab ((R2011b v7.13)).

### Supervised classification algorithms

In order to identify possible correlates of protection on the experimental data in S2–S4 Tables, we applied two types of classification techniques, discriminant analysis and logistic regression [47]. The most popular versions of these techniques rely on a linear relationship (i.e., linear discriminant analysis-LDA) between the correlates of protection and TB infection outcome of the macaques. Due to the relatively poor performance of these classical approaches, we also applied three other techniques that feature additional flexibility. The first is quadratic discriminant analysis (QDA), which can be appropriate if *quadratic* relationships exist between possible correlates and the TB infection outcome [47]. The last two techniques utilize a statistical technique, termed l_1_ regularization, that relies on constrained optimization to identify important variables in the model and hence minimize the misclassification error rate of the different models[47]. We refer to the last two models as penalized linear discriminant analysis (PLDA) and logistic regression. The cross-validation functions within the R package are used to select the regularization parameters for PLDA and logistic regression [48,49]. In addition to performing all techniques on the data sets, we also applied each technique to the data projected onto the first 3 principal components, which captured over 50% of variability in the data. Our goal in doing this was to try to improve performance by reducing the number of cytokine covariates.

Our goal is to predict TB infection outcome, a binary variable (1 for active; 0 for latent) and performances for the different methods are measured with a set of special metrics called sensitivity, specificity and misclassification error. Sensitivity is the rate at which active TB infections are correctly predicted (true positive rate), specificity is the rate at which latent TB infections are correctly predicted (true negative rate), and misclassification error rate is the overall prediction error rate [48]. To avoid reporting overly optimistic (biased due to over-fitting) results, we withhold 50% of the data when estimating the model parameters and test the fitted model on the withheld data. Henceforth, we refer to the withheld data as *test data* and the rest of the data used to estimate the model as *training data*. Results are qualitatively unchanged for a different number of macaques in the test dataset vs training set. Note also that data are considered by each of the four techniques to be cross-sectional, i.e., direct application of each method ignores temporal information.

### Unsupervised classification algorithms

Since we found that six macaques in the study were consistently incorrectly classified by our classification algorithms, we re-analyzed the blood data using unsupervised clustering algorithms (i.e., multidimensional scaling, Ward’s method and other hierarchical methods, and k-means)[47]. These algorithms attempt to find inherent groupings in the cytokine profiles, and hence could potentially identify a possible spectrum of latency rather than delineating between active and latent cases.

Multidimensional scaling (MDS) is a visualization technique that forms a two-dimensional representation of the macaques, where the location of each macaque in two-dimensional space is optimized so that macaques close together are most similar.

Complete and Average link hierarchical clustering, as well as Ward’s method are called “agglomerative” or bottom-up approaches, because they start with each macaque as its own cluster and then cluster them together until a stopping criteria is met. In contrast to agglomerative approaches, K-means is a top-down partitioning method, where initially all macaques belong to a single cluster. The clusters are then progressively split into smaller clusters (reviewed in [47]). One would expect to find consistent results between these different techniques, even though they take varying approaches to discovering macaque groupings. Wildly varying results across methods would indicate that the data feature high levels of noise that mask a possible structure between macaques.

### Computational model

Our hybrid agent-based model (ABM, labeled *GranSim*) tracks Mtb infection in 3 physiological compartments: lung (site of infection), draining lymph node (LN, site of generation of adaptive immunity) and blood (a measurable compartment, see Fig 2B for details). Our computational model captures single granuloma formation and function in the lung [30,31,32,33], while LN and blood compartments[28,29] represent dynamics of the whole body in response to infection. We simulate infection over a time span of 600 days, with rules and interactions solved on a 10 min time step which can be found at http://malthus.micro.med.umich.edu/GranSim/.

The lung environment represents a 2x2 mm section of lung parenchyma tissue. Details on lung initialization can be found in [30,31,32]. Cell types captured in the model are macrophages and T cells (or T lymphocytes). Macrophages transition between the following states: resting, activated, infected and chronically infected. T cells are represented by their functions: Tγ (i.e., IFNγ-producing T cells, necessary for macrophage activation), T_cyt_ (i.e., cytotoxic T cells) and T_reg_ (regulatory T cells). Two effector molecules are also tracked, namely tumor necrosis factor-α (TNF) and interleukin 10 (IL-10).

LN and blood dynamics are each captured by compartmentalized system of ordinary differential equations (ODEs) (see S1 Text for the equations and derivation). We updated our published multi-compartment computational models^34, 35^, in which we now track CD4+ and CD8+ T lymphocytes with different memory phenotypes (i.e., Naïve, Effector, Central and Effector Memory).

Two mechanisms new to *GranSim* are used herein: T cell recruitment and proliferation at the site of infection (described in detail in S2 Text—T cell recruitment and T cell proliferation in the lung).

### Lymph node and blood compartment

A great advantage of the *in silico* representation is that we can track both Mtb-specific and non Mtb-specific T cells (the *T cell dataset* does not discriminate between Mtb-specific and non-Mtb-specific cell types). The lymph node-blood dynamics are captured by 31 ODE equations with 21 independent parameters. Measure units are cell counts in the LN and cell/mm^3^ in blood. Details on the initial conditions of the model as well as parameter ranges are given in S8 and S9 Tables. Fig 2B illustrates the different T lymphocyte phenotypes tracked in the model and some assumptions regarding cell priming, trafficking and differentiation.

Our experimental data show that a single LN drains multiple granulomas forming in lungs [20,21,22]. We coarse-grain cell migration from the site of infection (*GranSim*) to a draining lymph node. Antigen presentation and priming occurring in LNs is driven by a proxy for antigen presenting cell dynamics (APCs, Eqn (1.1) in S1 Text), which is the count of macrophages in the lung that interacted with Mtb (M_Mtb_) at any time during infection (see [28] for details on the implementation). However, only a small fraction of cells (e.g., ~5–10%) that encounter Mtb in lungs is thought to be able to traffic and migrate to LNs [50,51,52,53,54]. Due to computational limitation, the lung compartment tracks formation and progression of a single granuloma and not multiple granulomas developing simultaneously. Thus, the M_Mtb_ count is an overestimate for the APC trafficking to the LN from a single granuloma. However, we use the M_Mtb_ count as a reasonable proxy for APC dynamics for the whole host. This assumption implies that we are calibrating the computational model as if the host is developing between 10 and 20 similar granulomas in the lungs (matching the ~5–10% cells that likely migrate to the LN upon infection) [25]. This allows us to use the *T cell dataset* to calibrate the computational model in the blood (since the blood data measured in that dataset reflect TB infection of a whole NHP), as well as in the lung by using our data on CFU per granuloma in NHPs [21,22]. Based on the above assumptions, when multiple granulomas are combined together to generate virtual NHPs and capture heterogenous granuloma outcomes within a single host, the *in silico* blood readouts for the whole host are computed as averages and medians (instead of sums) across all the granulomas sampled (see Figs 6 and 7 and S8 and S9 Figs). We are currently working on a computational platform where multiple granulomas can be simulated simultaneously and have potential to interact with each other, mimicking a whole lung infection dynamics.

Two T-cell phenotypes traffic between LN and blood, namely naïve and central memory cells (separate equations are described for CD4+ and CD8+ T cells, as well as for Mtb-specific and non-Mtb-specific, see S1 Text for details). Mtb-specific naïve T cells are primed by APCs in LNs to become precursor T cells, which proliferate and ultimately differentiate into Mtb-specific Central Memory and/or Mtb-specific Effector, depending on the strength of APC stimulation [55,56,57,58]. Mtb-specific Central Memory cells can be re-stimulated in LNs (similarly to Naïve) and become precursor again [59,60]. Mtb-specific Effector cells can differentiate into Mtb-specific Effector Memory cells [61]. All cells in the LN compartment (except APC and precursor) migrate into blood (as shown in Fig 2B).

We modeled Mtb-specific CD4+ T cell processes in the LN and blood compartments identical to how CD8+ T cells are modeled, with the exception of Mtb-specific Naïve CD8+ priming which is dependent of Mtb-specific Naïve CD4+ priming. We modeled non-Mtb-specific T cells (grey circles in Fig 2B) similarly to their respective Mtb-specific cell types (colored circles in Fig 2B) counter parts. However, non-Mtb-specific cells do not respond to antigen, therefore, no priming occurs in any of these cell populations in LNs and no precursor cells are generated. We assume that neither effector nor effector memory cells re-enter the LN after migrating into blood: they recirculate through blood and eventually migrate to lung (as shown in Fig 2B). The production of non-Mtb-specific effector cells was captured as a source term in blood and was calibrated to the *T cell dataset* prior to infection (S5 Table).

### Computation information

*GranSim* is an agent-based model implemented using the C++ programming language in conjunction with Boost libraries (distributed under the Boost Software License–available at www.boost.org). The graphical user interface (GUI) was built using the Qt framework (open-source, distributed under GPL–available at qt.digia.com), which allows us to display, track and plot different readouts of the *in silico* granuloma simulation in real-time. The lymph node and blood compartments are modeled together as an ordinary differential equation (ODE) system (as shown in Fig 2B). They are interfaced with *GranSim* by numerical ODE solvers implemented as part of the C++ platform all within our own code. The three-compartmental model can be used with or without GUI visualization and is cross-platform (Mac, Linux, Windows). Computational model simulations were performed on XSEDE’s OSG Condor pool and NERSC’s Edison Cray XC30. Initial ODE model calibration and analysis of the results were performed in Matlab, as well as all post-processing analysis of data generated by our *in silico* model. We use the min-max range NHP pre-infection data from the *T cell dataset* to establish ranges for the initial conditions for the blood ODE model (homeostasis, see S8 Table for details on the initial conditions). We assume that these levels represent a flow of cells constantly trafficking through LNs. Therefore, initial numbers of cells in the lung draining LN were calculated by dividing the number of those in the blood, by the number of LNs in the host (i.e., parameter *host_Ln* in S9 Table*)*. We also assumed a frequency of Mtb-specific Naïve T cells (parameter λ) between 0.001 and 0.00001 (i.e., [1e^-3^, 1e^-5^]) [62].

Most model parameter values used herein are taken from our previously published studies [28,29,30,31,53,63,64] and are listed in S9 Table. Additionally, we rely on uncertainty analysis techniques to efficiently explore the parameter space and inform on baseline behaviors of the system (uncertainty analysis—UA). Here we used Latin Hypercube Sampling (LHS, reviewed in [65]) for UA. The LHS algorithm is a stratified Monte Carlo sampling method without replacement [65]. It was used to generate 1,000 unique parameter sets, which are simulated in replication 10 times (a total of 10,000 *in silico* simulations). We varied 21 parameters/mechanisms in blood and LN compartments, as well as 8 initial conditions for Naïve, Effector, Effector Memory and Effector Memory CD4+ and CD8+ (see S8 and S9 Tables for the ranges used to generate the *in silico* granulomas and blood/LN dynamics). Parameters/mechanisms in *GranSim* have been updated to reflect latest knowledge and data (see [30] for details and http://malthus.micro.med.umich.edu/GranSim/) and were held fixed in our LHS experiments as blood and LN parameters were varied.

### The *in silico* dataset analysis

The *in silico* dataset comprises sets of 10,000 model simulations (i.e., 1,000 x 10 replications) of single granulomas coupled to LN and blood dynamics over a time span of 600 days post infection (step 3 in Fig 1). We analyzed the following readouts across the three compartments: blood compartment (16 variables), lymph node (15 variables), macrophage and T cell counts in the lung, as well as measures in the lung such as lesion size, TNF and IL-10 total levels, cytotoxic killing, and infected macrophage bursting rate (total of 48, see S11 Table for the complete list). We used the same time points from the experimental data (e.g., 9 time points from the *T cell dataset*, days 10, 20, 30, 42, 56, 90, 120, 150 and 180 post infection) and computational model simulations for direct comparison between the *in vivo* and *in silico* model outputs for the blood measures (step 2 in Fig 1). We extended the analysis up to 600 days to perform the scaling-to-host step, since we matched to NHPs that have been necropsied between 200 and 600 days post infection. Principal component analyses (PCA) were performed on the *in silico* dataset (step 4 in Fig 1), after the computational model was calibrated to the experimental data. We defined Mtb-specific frequencies for each T cell phenotype as the ratio between the number of Mtb-specific T cells over the total number of T cells.

### Model calibration

The computational model was calibrated with respect to i) CFU/granuloma dynamics, based on our recent NHP experimental data [21,22] (see S1 Table and step 2 in Fig 1), and on ii) blood T cell dynamics as measured in the *T cell dataset* (see S5 Table and step 2 in Fig 1). S8 and S9 Tables shows the ranges used to generate our *in silico dataset* of 10,000 granulomas (step 3 in Fig 1). The 10,000 model simulations returned CFU dynamics as well as T cell dynamics in the blood that we compare to the NHP experimental data (as shown in Figs 3 and 4). Since the *T cell dataset* measured total T cell populations rather than Mtb-specific subsets, we summed Mtb-specific and non Mtb-specific *in silico* cell predictions to match our experimental data. Due to the large variability in the *T cell dataset*, we did not superimpose strict criteria for model calibration. The computational model was considered to be adequately calibrated if minimum, mean, median and maximum *in silico* trajectories were within the longitudinal data measured in the experimental settings.

Only 12 NHPs (of the 28 in the datasets used for classification) have both blood and lung data available, so we used these 12 NHPs for the initial model calibration (Fig 4). We then merged both the lung data on CFU of the 43 NHPs with the blood data of the 12 NHPs to see if all the virtual NHPs have blood dynamics within the experimental ranges (Fig 4). The 12 NHPs are biased towards latent (9) vs active TB (3) and limited to 6 months (while the lung experimental data are available up to 600 days). Fig 5 has the T cell Memory phenotype data shown as frequencies of T cell producing any cytokine in response to ESAT6/CFP10 stimulation.

For the reasons outlined above and since time courses of NHP blood experimental data are not available to match our Mtb-specific T cell frequencies predictions (step 6 in Fig 1), Figs 7, S8 and S9 only show *in silico* time courses.

### Building virtual NHPs for scaling-to-*host in silico* biomarker predictions

To allow our model to be directly comparable to NHP and human data that track infection progression, we developed a method to scale single granuloma outcomes in lungs to host level readouts in blood. The virtual NHP generation approach uses a different set of NHP data compared to the machine learning approach (step 5 in Fig 1). We guide the generation of virtual NHPs by replicating granuloma heterogeneity and variability in the lung up to 600 days post infection (i.e., # of granuloams and CFU/granuloma in S1 Table). We then analyze the *in silico* blood dynamics to make predictions on potential biomarkers for infection outcome. The complete protocol, illustrated in Fig 6, comprises the following steps: i) select the number of granulomas to replicate upon infection over time in each known NHP from our NHP experimental data (see S1 Table), ii) mimic CFU/granuloma values by sampling only granulomas that have similar CFU burden from our repository of 10,000 *in silico* granulomas (based on Figs 3 and 4, the computational model readouts are within the range of variability measured by experimental data in the lung and in the blood, thus the sampling is warranted), iii) collect *in silico* blood readouts predicted for each granuloma, and then average them across all granulomas, iv) repeat steps ii) and iii) K times (to capture within host variability, we used K = 1,000) and generate statistics (e.g., means, medians, standard deviations) on the K replications to recapitulate *in silico* blood data on the single virtual host, v) repeat steps i)-iv) for all the NHPs in the dataset.

For example, NHP 21710 (latent TB, row 14 in S1 Table) has 12 granulomas; 8 of them are sterile and 4 have the following CFU burdens: 38, 142, 38 and 1133. Virtual NHP 21710 was built by sampling 12 *in silico* granulomas from our repository at the day NHP 21710 was necropsied (i.e., 370 days post infection). For sterile granulomas, we use the criteria of CFU<1 per granuloma, and for the non-sterile granulomas we sample from subsets of *in silico* granulomas that fell within a ±α range of the NHP experimental data (Fig 6). We used α = 10%. Numbers of *in silico* granulomas that satisfy our condition for sterile granulomas (i.e. CFU<1) at day 370 post infection is 2257 out of 10,000. For the first non-sterile granuloma of NHP 21710 (CFU = 38), we first select *in silico* granulomas with CFU in the ±10% range, namely [30,46] (i.e., 66 granulomas), and then randomly choose one without replacement (thus the third granuloma, which has the same CFU, will not be assigned to the *in silico* granuloma selected for the first one). The second granuloma will be selected from the range [113,170] (i.e., 12 granulomas), and so on. Once all the *in silico* granulomas have been sampled, we average the blood readouts across all the simulated granulomas (12 granulomas for NHP 21710) and then compute the values for total T cell levels as well as for the Mtb-specific T cell frequencies in the blood. We repeat the same procedure K times (K = 1000) for the same virtual NHP to account for granuloma outcome variability and generate statistics (mean, median, standard deviations) for the variables used to correlate *in silico* blood time courses to infection outcome (e.g., total T cell levels as well as Mtb-specific T cell frequencies, see Fig 6 for details).

We applied the scaling to host method to 43 NHPs that have been classified as either latent (20 NHPs) or active (23 NHPs) TB, and that have data on numbers of granulomas in lung and CFU/granuloma for each granuloma (highlighted in yellow in S1 Table). Once all the blood readout statistics have been computed on the 43 virtual NHPs, we generated trajectories (see Figs 7, S8 and S9) by grouping active vs latent virtual NHPs and we test if any of them are significantly different (t-test) over time and likely predictive of infection outcome (step 6 in Fig 1). Figs 7, S8 and S9 show how we used *in silico* predictions for the time courses of total CD4+ and CD8+ T cell levels, as well as Mtb-specific T cell frequencies to predict the virtual NHPs infection outcome, using the clinical known classification between latent and active TB groups from S1 Table.

## Supporting Information

S1 Text Supplementary Materials and Methods. Mathematical Model describing cell population dynamics in the blood and lymph node compartments of the computational model described in Fig 2B.
(DOCX)

S2 Text Supplementary Materials and Methods. T cell recruitment and T cell proliferation in the lung.
(DOCX)

S1 Fig Representative flow cytometry plots. (2712- 6months post infection-stimulated with ESAT-6 (A-D) or P&I (E)) outlining gating strategies employed in the analysis T cells in PBMC. (A) Singlets were gated based on Forward scatter height (FSC-H) and area (FSC-A). From the singlets, (B) Lymphocytes were selected based on SSC and FSC (i.e., size and granularity). CD4 or CD8 T cells (C) were gated on the lymphocyte population. From either CD4 or CD8 T cells memory subsets (D) were selected based on CD45RA and CD27 markers as follows: CD45RA+CD27+ as Naïve, CD45RA-CD27+ as Central memory, CD45RA-CD27- as Effector memory and CD45RA+CD27- as Effector or terminally differentiated. Cytokine producing CD4 or CD8 T cells or memory subsets were gated as shown (E). Arrow indicates sequence of gating.
(TIF)

S2 Fig ROC, sensitivity and specificity of the results of supervised classification algorithms applied to the blood data of the 28 NHPs. The performances of the binary classification algorithms shown in Table 2 have been measured on the single and memory cytokine datasets by calculating their receiving operating characteristic (ROC) curves (Panels A and B). The area under the curve (AUC) and misclassification error values ere shown in Panels C and D. The script to generate the ROCs have been written in R, using the library “ROCR” and the performance function with true (i.e., tpr) and false positive rates (i.e., fpr) arguments for the cost function (e.g., performance(pred,"tpr","fpr")). The cost associated with tpr and fpr is the same.
(TIF)

S3 Fig Biomarker discovery on the *in silico* data. *(A-C)*: Principal Component Analysis (PCA) performed only on Mtb-specific variables in the blood compartment from the same repository of 10,000 *in silico* granuloma simulations used to generate Fig 4. *(A)*: Pareto plot of the top 10 PCAs. We can reach ~70% of explained variance by summing the top 2 PCAs. *Panel B***:** scatterplot of PCA1 and PCA2. *Panel C***:** biplot associated to Panel B, with categories listed on the four quadrants. Each category is listed as Mtb-specific T cell phenotype at a certain time point post infection. For example “*E8 42”* refers to Effector CD8+ T cells at day 42 post infection. (Other T cell phenotypes shown: CM [central memory]).
(TIF)

S4 Fig Principal Component Analysis (PCA) applied to the *in silico* data generated by the 3-compartmental model. Blood and Lung readouts (49 readouts total). (A)-(C): scatter plots of the 1^st^ principal component versus the 2^nd^, 3^rd^ and 4^th^ principal component, respectively. (D)-(E): scatter plots of the 2^nd^ principal components versus the 3^rd^ and 4^th^ principal components. (F): scatter plot of the 3^rd^ and 4^th^ principal components.
(TIF)

S5 Fig Biplots associated to S3 Fig. See S11 Table for details on the labels of the scores. The number after the underscore sign refer to the day after infection on which that variable as been measured. We plot the top 4 principal components because they explain ~60 of the variability. (A)-(C): biplots of the scores associated with the scatter plots of the 1^st^ principal component versus the 2^nd^, 3^rd^ and 4^th^ principal component (as shown in S4 Fig, panels (A)-(C)), respectively. (D)-(E): biplots of the scores associated with the scatter plots of the 2^nd^ principal components versus the 3^rd^ and 4^th^ principal components (as shown in S4 Fig, panels (D)-(E)). (F): biplot of the scores associated with the scatter plot of the 3^rd^ and 4^th^ principal components (as shown in S4 Fig, panel (F)).
(TIF)

S6 Fig Biomarker discovery on the *in silico* data. Each panel shows the same repository of 10,000 *in silico* granuloma simulations coupled to the blood and LN dynamics used to generate Figs 3 and 4. Each point on the plots represents one *in silico* granuloma. Here we couple information from both the blood (x-axis) and the lung (y-axis). The y-axis represents CFU/granuloma, while the x-axis is the ratio of Mtb-specific vs non Mtb-specific Effector CD4+ cell levels in the blood at day 167 post infection. Both axis are displayed on a log scale. Panels B and F are used in S7 Fig (panels C and D) for detailed studies. (A)-(D): scatter plots of CFU per granuloma (y-axis) versus Mtb-specific frequencies of different CD4+ T cell phenotypes (i.e., Naïve, Effector, Central Memory and Effector Memory). (E)-(H): scatter plots of CFU per granuloma (y-axis) versus Mtb-specific frequencies of different CD8+ T cell phenotypes (i.e., Naïve, Effector, Central Memory and Effector Memory).
(TIF)

S7 Fig Biomarker discovery on the *in silico* data. (A-D): Scatter plots of the same repository of 10,000 *in silico* granuloma simulations coupled to the blood and LN dynamics used to generate Figs 3 and 4. Each point on the plots represents one *in silico* granuloma. Here we couple information from both the blood (x-axis) and the lung (y-axis). The y-axis represents CFU/granuloma, while the x-axis is the Mtb-specific frequency of Effector CD4+ (A—day 140 / C—day 167) and CD8+ (B—day 140 / D—day 167) cell levels in the blood (A—day 140 / B—day 167). Mtb-specific frequency is calculated by dividing the number of Mtb-specific cells over the total T cells, within each specific phenotype. So, for example the values on the x axis of Panel D are calculated by dividing Mtb-specific Effector CD4+ T cell counts by the total Effector CD4+ T cell counts. Both axis are displayed on a log scale. The horizontal black lines are located at 100 CFU per granuloma and they separate granuloma clusters emerging from the 10,000 *in silico* simulations as either *low* or *high* CFU granulomas. Vertical red lines are the Mtb-specific frequency thresholds suggested in each panel to predict granuloma with *low* versus *high* CFU. We chose these two time points (i.e., day 140 and day 167) as they displayed the best separation between granuloma clusters with *low* versus *high* CFU burden. All other Mtb-specific frequencies that can be calculated on the *in silico* data (namely, naïve, effector, central and effector memory ratios for CD4+ and CD8+ T cells) and plotted against CFU/granuloma at the latest time point measured in the memory dataset (i.e., day 167 post infection) are shown in S6 Fig. There is a small number of granulomas that do not fall into either these groups, (i.e., where high CFU is associated with low Mtb-specific T cell frequencies, false positive group if we use the frequency threshold as a biomarker).
(TIF)

S8 Fig Scaling to host infection outcome predictions–Total T cells and frequencies. Trajectories over 600 days of T cell levels, both total and Mtb-specific, as well as Mtb-specific T cell frequencies. The data shown have been generated following steps illustrated in Fig 6, as well as in the Materials and Methods section. The 43 virtual NHPs have been classified based on known clinical outcome and are displayed in all the panels as mean +/- 2x(standard error). The asterisks show significant (p<0.05) student t-test between the two trajectories at the same time point. *Panel A*: Total T cell levels. *Panel B*: Total CD4+ T cell levels. *Panel B*: Total Mtb-specific T cell levels. *Panel C*: Total non Mtb-specific T cell levels. *Panel D*: Total non Mtb-specific CD4+ T cell levels. *Panel E*: Total non Mtb-specific CD8+ T cell levels. *Panel F*: Frequency of Mtb-specific Naïve CD4+ T cells. *Panel G*: Frequency of Mtb-specific Central Memory CD4+ T cells. *Panel H*: Frequency of Mtb-specific Naïve CD8+ T cells. *Panel I*: Frequency of Mtb-specific Central Memory CD8+ T cells.
(TIF)

S9 Fig Scaling to host infection outcome predictions–Ratios of Mtb-specific CD4+ T cell phenotypes. Trajectories over 600 days of ratios of Mtb-specific CD4+ T cell levels. The data shown have been generated following steps illustrated in of 6, as well as in the Materials and Methods section. The 43 virtual NHPs have been classified based on known clinical outcome and are displayed in all the panels as mean +/- 2x(standard error). The asterisks show significant (p<0.05) student t-test between the two trajectories at the same time point. *Panel A*: Ratio of Effector Memory over Central Memory Mtb-specific CD4+ T cells. *Panel B*: Ratio of Effector over Central Memory Mtb-specific CD4+ T cells.
(TIF)

S1 Table Non-human primate data on infection outcome classification, number of granulomas and CFU/granuloma. (XLS)

S2 Table Single cytokine dataset (blood). (XLSX)

S3 Table Multiple cytokine dataset (blood). (XLS)

S4 Table Memory single cytokines dataset (blood). (XLSX)

S5 Table T cell dataset (blood). (XLS)

S6 Table ESAT6-CFP10 memory T cell dataset (blood). (XLS)

S7 Table Unsupervised classification algorithms results. (PDF)

S8 Table Parameter values and ranges for the initial conditions of the computational model. (PDF)

S9 Table All other parameter values and ranges of the computational model. (PDF)

S10 Table Statistics of S6 Fig. (DOC)

S11 Table List of *in silico* data readouts. (PDF)
