# Supplementary material for: Computational and Empirical Studies Predict Mycobacterium tuberculosis-Specific T Cells as a Biomarker for Infection Outcome
Source: PLoS Comput Biol. 2016 Apr 11;12(4):e1004804. doi: 10.1371/journal.pcbi.1004804 (PMC4827839; doi:10.1371/journal.pcbi.1004804)
Supplement: S1 Text — Mathematical Model describing cell population dynamics in the blood and lymph node compartments of the computational model described in Fig 2B. (DOCX) [file pcbi.1004804.s001.docx]

**S1 TEXT**

to the manuscript

**Computational and empirical studies predict *Mycobacterium tuberculosis*-specific T cells as a biomarker for infection outcome**

Simeone Marino^1*^, Hannah P. Gideon^2*^, Chang Gong^1^ , Shawn Mankad^3^, John T. McCrone^1^, Philana Ling Lin^4^ Jennifer J. Linderman^5^, JoAnne L. Flynn^2^, Denise E. Kirschner^1^

^1^ Department of Microbiology and Immunology, University of Michigan Medical School, Ann Arbor, Michigan, USA ^2^ Department of Microbiology and Molecular Genetics, University of Pittsburgh, Pittsburgh, PA, USA. ^3^ Robert H. Smith School of Business. University of Maryland, College Park, MD. USA ^4^Department of Pediatrics, Children’s Hospital of the University of Pittsburgh of UPMC, Pittsburgh, PA, USA. ^5^ Department of Chemical Engineering, University of Michigan, Ann Arbor, MI, USA

**Mathematical Model describing cell population dynamics in the blood and lymph node compartments of the computational model described in Figure 2B**

The next section describes in detail how we developed an ordinary differential equation model for CD4+ Mtb-specific T cells dynamics.

Measure units are cell count in the lymph compartment and cell/mm^3^ in the blood compartment. The term α , represents the volume of blood in μL and is used for scaling cells when they traffic between the blood compartment and the lymph compartment.

Antigen presentation and priming in the lymph node compartment is driven by the following equation

$$\frac{dAPC}{dt}=-\mu_{5}APC$$

which tracks antigen presenting cells (APCs) based on the number of macrophages in the lung compartment that interacted with Mtb (M_Mtb_) at any time during infection. This assumes that infected macrophages represent a proportion of dendritic cells that would leave the lung and travel to draining LNs.

As the number of M_Mtb_ increases at the site of infection, the integer increment(s) is added as new initial condition for APC Eqn. before each iteration. If the number of M_Mtb_ doesn’t increase or decreases, the APC number decreases following the exponential decay, at the rate μ_5_.

Naïve T cells (Eqn. ) are recruited to the lymph node at a rate (*k_1_*) dependent on cytokine production in the lymph node. Since we do not track cytokines in the lymph node model, we use APC also as a proxy for cytokine production (modeled as a Michaelis-Menten term in Eqn. ). Other terms included basal influx ($\xi_{1}$) and efflux ($\xi_{2}$), as well as mass action priming to precursor cells (*k_2_*).

$$\frac{dN_{4}^{LN}}{dt}=\alpha\left(k_{1}N_{4}^{B}\left(\frac{APC}{APC+hs_{1}}\right)+\xi_{1}N_{4}^{B}\right)-\xi_{2}N_{4}^{LN}-k_{2}N_{4}^{LN}APC$$

Precursor CD4+ T cells (Eqn. ) are generated through priming of Mtb-specific naïve T cells as wells as through re-activation of Mtb-specific central memory T cells; both processes are expressed as mass action terms. Proliferation is modeled as logistic growth.

$$\begin{matrix} \frac{dP_{4}^{LN}}{dt}=APC\left(k_{2}N_{4}^{LN}+k_{3}CM_{4}^{LN}\right)+k_{4}P_{4}^{LN}\left(1-\left(\frac{P_{4}^{LN}}{\rho_{1}}\right)\right)\left(\frac{APC}{APC+hs_{4}}\right) \\ -k_{5}P_{4}^{LN}\left(\frac{APC}{APC+hs_{5}}\right)-k_{6}P_{4}^{LN}\left(1-\left(\frac{APC}{APC+hs_{5}}\right)\right)-\mu_{6}P_{4}^{LN} \end{matrix}$$

A Michaelis-Menten term based on antigen stimulation (APC levels) was used to adjust proliferation (k_4_) and differentiation rates (k_5_ and k_6_). The likelihood of precursor cells differentiating into effector cells is directly proportional to the amount of antigen stimulation (k_5_). The opposite assumption was applied to the likelihood of precursor cells differentiating into central memory (k_6_). A death term (μ_6_) ensured that the precursor population did not persist in the absence of infection. No precursor population exits the lymph node. Effector CD4+ T cells are modeled in Eqn. .

Terms in the equation include efflux to the blood ($\xi_{3}$), and a linear differentiation to the effector memory T cell population (*k_7_*). We assumed that no effector T cells die in the lymph node (they can die in the blood before entering the lung) and that no migration occurs directly from the blood.

Similar to naïve cells, central memory T cells (Eqn. ) are recruited to the lymph node (k_8_) in addition to a basal influx rate ($\xi_{4}$). Other terms include differentiation from precursor cells (k_6_), reactivation to precursor cells (k_3_) and efflux into the blood ($\xi_{4}$). Given their relatively long lifespan compared to the length of the in silico simulation (i.e., 200 days) we do not have a death term in Eqn. .

Effector memory cell formation is described in Eqn. . A linear term captures the differentiation of CD4+ effector T cells into CD4+ effector memory (k_7_). The last term represented efflux to the blood ($\xi_{6}$). Given their long life span, we do not account for cell death in Eqn. . Like effector T cells, effector memory T cells do not enter the lymph node directly from the blood.

$$\frac{dEM_{4}^{LN}}{dt}=k_{7}E{}_{4}^{LN}-\xi_{6}EM_{4}^{LN}$$

For the blood compartment we track 4 different T cell Mtb-specific phenotypes. The Mtb-specific naïve CD4+ T cell blood population is modeled by Eqn. . We have terms for a constant source supplied from the thymus (multiplied by the Mtb-specific frequency λ, i.e. $\lambda s_{N_{4}}$) to track specific and non-specific cells, migration from the lymph node ($\xi_{2}$), extra recruitment to the lymph node (k_1_), migration to the lymph node ($\xi_{1}$), and death (μ_8_).

$$\frac{dN_{4}^{B}}{dt}=\lambda s_{N_{4}}+\alpha^{-1}\xi_{2}N_{4}^{LN}-k_{1}N_{4}^{B}\left(\frac{APC}{APC+hs_{1}}\right)-\xi_{1}N_{4}^{B}-\mu_{8}N_{4}^{B}$$

The values for $s_{N_{4}}$ and μ_8_ (as well as $s_{N_{8}}$ and μ_9_ later for CD8+ T cells) are chosen to maintain equilibrium in the total Naïve T cell populations (based on the initial conditions taken from the NHP blood data).

Eqn. describes effector CD4+ T cells dynamics in the blood with two terms: migration from the lymph node ($\xi_{3}$) and death. Effector T cells are recruited to the lung during each ABM time step. This loss was implicated as a change in the initial conditions before each ODE time step

$\frac{dE_{4}^{B}}{dt}=\alpha^{-1}\xi_{3}E_{4}^{LN}-\mu_{1}E_{4}^{B}$.

Central memory cells in the blood (Eqn. ) migrate from ($\xi_{5}$) and to the lymph node ($\xi_{4}$). Central memory cells are not recruited to the site of infection.

$$\frac{dCM_{4}^{B}}{dt}=\alpha^{-1}\xi_{5}CM_{4}^{LN}-\xi_{4}CM_{4}^{B}-k_{8}CM_{4}^{B}\left(\frac{APC}{APC+hs_{8}}\right)$$

Effector memory cells in the blood (Eqn.) are modeled by two terms: migration from the lymph node ($\xi_{6}$) and death. Similar to effector cells these were recruited to the site of infection.

$$\frac{dEM_{4}^{B}}{dt}=\alpha^{-1}\xi_{6}EM_{4}^{LN}-\mu_{2}EM_{4}^{B}$$

**Lymph Node and Blood compartment equations for Mtb-specific CD8+ cells**

We modeled dynamics of Mtb-specific CD8+ T cell processes in the lymph node and blood identical to how we did for CD4+ T cells, with the exception of few terms and different parameter values. One such exception is the priming of Mtb-specific naïve CD8+ T cells in the lymph node, which is affected by cytokines released by activated CD4+ T cells in the lymph node. This is modeled by a Michaelis-Menten term including activated CD4+ T effector cells and a weighted term for CD4+ precursor T cells (as shown in the equations and , respectively Mtb-specific naïve and precursor CD8+ T cells in the LN).

The equations for Mtb-specific CD8+ T cells in the lymph node and blood compartments are described below.

*Effector CD8+ - LN*

$\frac{dE_{8}^{LN}}{dt}=k_{14}P_{8}^{LN}\left(\frac{APC}{APC+hs_{14}}\right)-\xi_{9}E_{8}^{LN}-k_{16}E_{8}^{LN}$ (1.13)

*Central Memory CD8+ - LN*

$\begin{matrix} \frac{dCM_{8}^{LN}}{dt}=\alpha\left(k_{17}CM_{8}^{B}\left(\frac{APC}{APC+hs_{17}}\right)+\xi_{10}CM_{8}^{B}\right)+ \\ k_{15}P_{8}^{LN}\left(1-\left(\frac{APC}{APC+hs_{14}}\right)\right)-k_{12}CM_{8}^{LN}APC-\xi_{11}CM_{8}^{LN} \end{matrix}$ (1.14)

*Effector Memory CD8+ - LN*

$\frac{dEM_{8}^{LN}}{dt}=k_{16}E{}_{8}^{LN}-\xi_{12}EM_{8}^{LN}$ (1.15)

*Naïve CD8+ - Blood*

$\frac{dN_{8}^{B}}{dt}=\lambda s_{N_{8}}+\alpha^{-1}\xi_{8}N_{8}^{LN}-k_{10}N_{8}^{B}\left(\frac{APC}{APC+hs_{10}}\right)-\xi_{7}N_{8}^{B}-\mu_{9}N_{8}^{B}$ (1.16)

*Effector CD8+ - Blood*

$\frac{dE_{8}^{B}}{dt}=\alpha^{-1}\xi_{9}E_{8}^{LN}-\mu_{3}E_{8}^{B}$ (1.17)

As with CD4+ effector cells in the blood, recruitment to the site of infection was modeled by changing the initial conditions before each ODE time step.

*Central Memory CD8+ - Blood*

$\frac{dCM_{8}^{B}}{dt}=\alpha^{-1}\xi_{11}CM_{8}^{LN}-\xi_{10}CM_{8}^{B}-k_{17}CM_{8}^{B}\left(\frac{APC}{APC+hs_{17}}\right)$ (1.18)

*Effector Memory CD8+ - Blood*

$\frac{dEM_{8}^{B}}{dt}=\alpha^{-1}\xi_{12}EM_{8}^{LN}-\mu_{4}EM_{8}^{B}$ (1.19)

**Non Mtb-specific CD4+ and CD8+ lymphocytes**

Our computational model keeps track of non Mtb-specific T cells similarly to their respective Mtb-specific (antigen specific) counter parts. However, non Mtb-specific T cells do not respond to antigen, therefore, no priming occurs in any cell population and no precursor cells are generated. Also, since we assume neither effector nor effector memory T cells enter the lymph compartment from the blood, we do not model effector or effector memory cell populations within the lymph node compartment (as shown in Figure 1B). The production of the non-TB specific effector cells was modeled as a source term in the blood compartment and was included to meet the assumption that the pre-infection data describes homeostasis. The equations for non-Mtb-specific CD4+ and CD8+ T cells are shown below. Moreover, including non-Mtb-specific cells at the site of infection makes model predictions more realistic due to the total cell numbers more accurately reflecting the actual numbers in blood..

*Naïve CD4+ non Mtb-specific - LN*

$\frac{dN_{nc4}^{LN}}{dt}=\alpha\left(k_{1}N_{nc4}^{B}\left(\frac{APC}{APC+hs_{1}}\right)+\xi_{1}N_{nc4}^{B}\right)-\xi_{2}N_{nc4}^{LN}$ (1.20)

*Central Memory CD4+ non Mtb-specific - LN*

$\frac{dCM_{nc4}^{LN}}{dt}=\alpha\left(k_{8}CM_{nc4}^{B}\left(\frac{APC}{APC+hs_{8}}\right)+\xi_{4}CM_{nc4}^{B}\right)-\xi_{5}CM_{nc4}^{LN}$ (1.21)

*Naïve CD4+ non Mtb-specific - Blood*

$\frac{dN_{nc4}^{B}}{dt}=(1-\lambda)s_{N_{4}}+\alpha^{-1}\xi_{2}N_{nc4}^{LN}-k_{1}N_{nc4}^{B}\left(\frac{APC}{APC+hs_{1}}\right)-\xi_{1}N_{nc4}^{B}-\mu_{8}N_{nc4}^{B}$ (1.22)

*Effector CD4+ non Mtb-specific - Blood*

$\frac{dE_{nc4}^{B}}{dt}=s_{E_{nc4}}-\mu_{1}E_{nc4}^{B}$ (1.23)

As non Mtb-specific effector cells in the blood must be produced somewhere in the body, they are modeled as source and a death rate equal to that of their Mtb-specific counterparts

*Central Memory CD4+ non Mtb-specific - Blood*

$\frac{dCM_{nc4}^{B}}{dt}=\alpha^{-1}\xi_{5}CM_{nc4}^{LN}-\xi_{4}CM_{nc4}^{B}-k_{8}CM_{nc4}^{B}\left(\frac{APC}{APC+hs_{8}}\right)$ (1.24)

*Effector Memory CD4+ non Mtb-specific - Blood*

$\frac{dEM_{nc4}^{B}}{dt}=s_{EM_{nc4}}-\mu_{2}EM_{nc4}^{B}$ (1.25)

As was the case with non Mtb-specific cells, CD8+ T cell processes are modeled identical to their CD4+ counterparts.

*Naïve CD8+ non Mtb-specific - LN*

$\frac{dN_{nc8}^{LN}}{dt}=\alpha\left(k_{10}N_{nc8}^{B}\left(\frac{APC}{APC+hs_{10}}\right)+\xi_{7}N_{nc8}^{B}\right)-\xi_{8}N_{nc8}^{LN}$ (1.26)

*Central Memory CD8+ non Mtb-specific - LN*

$\frac{dCM_{nc8}^{LN}}{dt}=\alpha\left(k_{17}CM_{nc8}^{B}\left(\frac{APC}{APC+hs_{17}}\right)+\xi_{10}CM_{nc8}^{B}\right)-\xi_{11}CM_{nc8}^{LN}$ (1.27)

*Naïve CD8+ non Mtb-specific - Blood*

$\frac{dN_{nc8}^{B}}{dt}=(1-\lambda)s_{N_{4}}+\alpha^{-1}\xi_{8}N_{nc8}^{LN}-k_{10}N_{nc8}^{B}\left(\frac{APC}{APC+hs_{10}}\right)-\xi_{7}N_{nc8}^{B}-\mu_{9}N_{nc8}^{B}$ (1.28)

*Effector CD8+ non Mtb-specific - Blood*

$\frac{dE_{nc8}^{B}}{dt}=s_{E_{nc8}}-\mu_{3}E_{nc8}^{B}$ (1.29)

*Central Memory CD8+ non Mtb-specific - Blood*

$\frac{dCM_{nc8}^{B}}{dt}=\alpha^{-1}\xi_{11}CM_{nc8}^{LN}-\xi_{10}CM_{nc8}^{B}-k_{17}CM_{nc8}^{B}\left(\frac{APC}{APC+hs_{17}}\right)$ (1.30)

*Effector Memory CD8+ non Mtb-specific - Blood*

$\frac{dEM_{nc8}^{B}}{dt}=s_{EM_{nc8}}-\mu_{4}EM_{nc8}^{B}$ (1.31)
