# Supplementary material for: Computational and Empirical Studies Predict Mycobacterium tuberculosis-Specific T Cells as a Biomarker for Infection Outcome
Source: PLoS Comput Biol. 2016 Apr 11;12(4):e1004804. doi: 10.1371/journal.pcbi.1004804 (PMC4827839; doi:10.1371/journal.pcbi.1004804)
Supplement: S2 Text — T cell recruitment and T cell proliferation in the lung. (DOCX) [file pcbi.1004804.s002.docx]

**TEXT S2** to the manuscript

**Computational and empirical studies predict Mycobacterium tuberculosis-specific T cells as a biomarker for infection outcome**

Simeone Marino^1*^, Hannah P. Gideon^2*^, Chang Gong^1^ , Shawn Mankad^3^, John T. McCrone^1^, Philana Ling Lin^4^ Jennifer J. Linderman^5^, JoAnne L. Flynn^2^, Denise E. Kirschner^1^

^1^ Department of Microbiology and Immunology, University of Michigan Medical School, Ann Arbor, Michigan, USA ^2^ Department of Microbiology and Molecular Genetics, University of Pittsburgh, Pittsburgh, PA, USA. ^3^ Robert H. Smith School of Business. University of Maryland, College Park, MD. USA ^4^Department of Pediatrics, Children’s Hospital of the University of Pittsburgh of UPMC, Pittsburgh, PA, USA. ^5^ Department of Chemical Engineering, University of Michigan, Ann Arbor, MI, USA

*These authors contributed equally to this work (co–first authors).

**T cell recruitment and T cell proliferation in the lung**

We use a subset of the blood equations (namely Effector and Effector Memory T cells, CD4+ and CD8+ T cells, both Mtb-specific and non Mtb-specific ) to compute the number of cells available in the blood for recruitment to the lung during infection. T cell recruitment occurs at each vascular source. First the program confirms specific cytokine and chemokine threshold conditions are fulfilled for each T cell phenotype (i.e., Tγ, Tcyt and Treg) and if there is at least one cell available to recruit for each phenotype. Then, depending on how many of these conditions are satisfied, the recruitment probabilities are obtained by normalizing the corresponding blood concentrations (each recruitment probability is proportional to the blood concentration, across phenotypes and Mtb-specific vs non Mtb-specific). The term α in S9 Table is used for scaling cell trafficking between the blood compartment and the lymph compartment and represents the volume of blood in μL (see S9 Table). The values in the blood are converted from concentrations to cell numbers by multiplying by α, the scaling factor for the compartment flow.

Once a T cell is recruited, the corresponding concentration is subtracted from the current numerical solution of the differential equation and the recruitment procedure is repeated for the next vascular source until all are checked. In the lung, only Mtb-specific T cells can proliferate, regardless of their phenotype (they all are fully effector T cells). Non Mtb-specific T cells in the lung do not proliferate or produce any cytokines. Proliferation occurs by antigen-dependent stimulation. If a Mtb-specific T cell resides in the proximity (i.e., Moore neighborhood) of a macrophage that has been exposed to Mtb in its lifespan, the proliferation clock starts and two parameters define the speed and magnitude of the division: doubling time (e.g., *proliferationTime*, see S9 Table) and maximum number of divisions (*maxDivisions*, see S9 Table).
