# Supplementary figures and images for: Computational and Empirical Studies Predict Mycobacterium tuberculosis-Specific T Cells as a Biomarker for Infection Outcome
Source: PLoS Comput Biol. 2016 Apr 11;12(4):e1004804. doi: 10.1371/journal.pcbi.1004804 (PMC4827839; doi:10.1371/journal.pcbi.1004804)

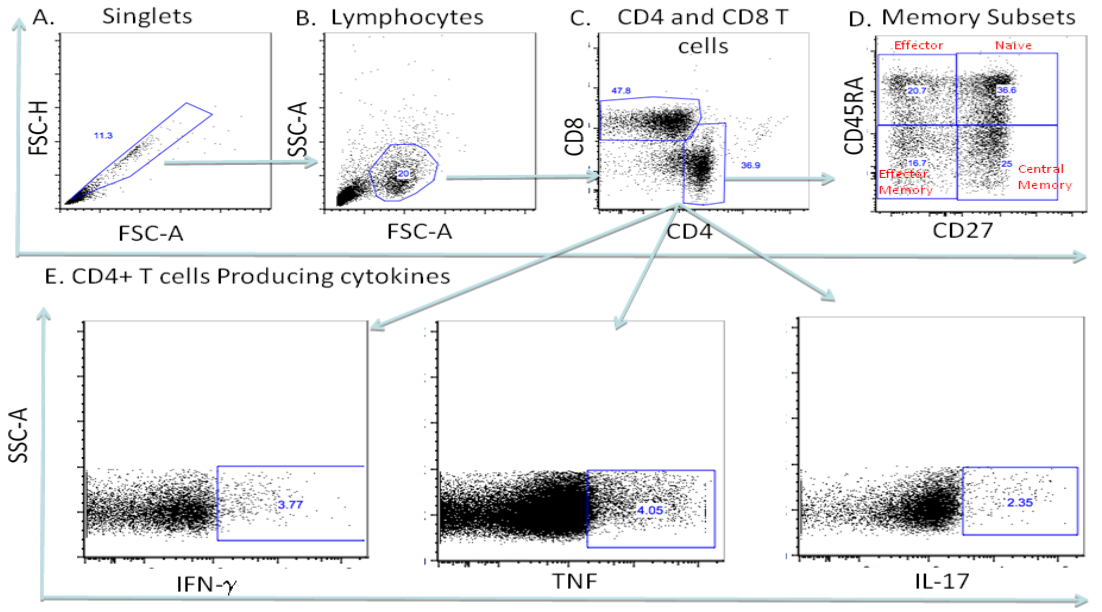

Supplement: S1 Fig — (2712- 6months post infection-stimulated with ESAT-6 (A-D) or P&I (E)) outlining gating strategies employed in the analysis T cells in PBMC. (A) Singlets were gated based on Forward scatter height (FSC-H) and area (FSC-A). From the singlets, (B) Lymphocytes were selected based on SSC and FSC (i.e., size and granularity). CD4 or CD8 T cells (C) were gated on the lymphocyte population. From either CD4 or CD8 T cells memory subsets (D) were selected based on CD45RA and CD27 markers as follows: CD45RA+CD27+ as Naïve, CD45RA-CD27+ as Central memory, CD45RA-CD27- as Effector memory and CD45RA+CD27- as Effector or terminally differentiated. Cytokine producing CD4 or CD8 T cells or memory subsets were gated as shown (E). Arrow indicates sequence of gating. (TIF) [file pcbi.1004804.s003.tif]

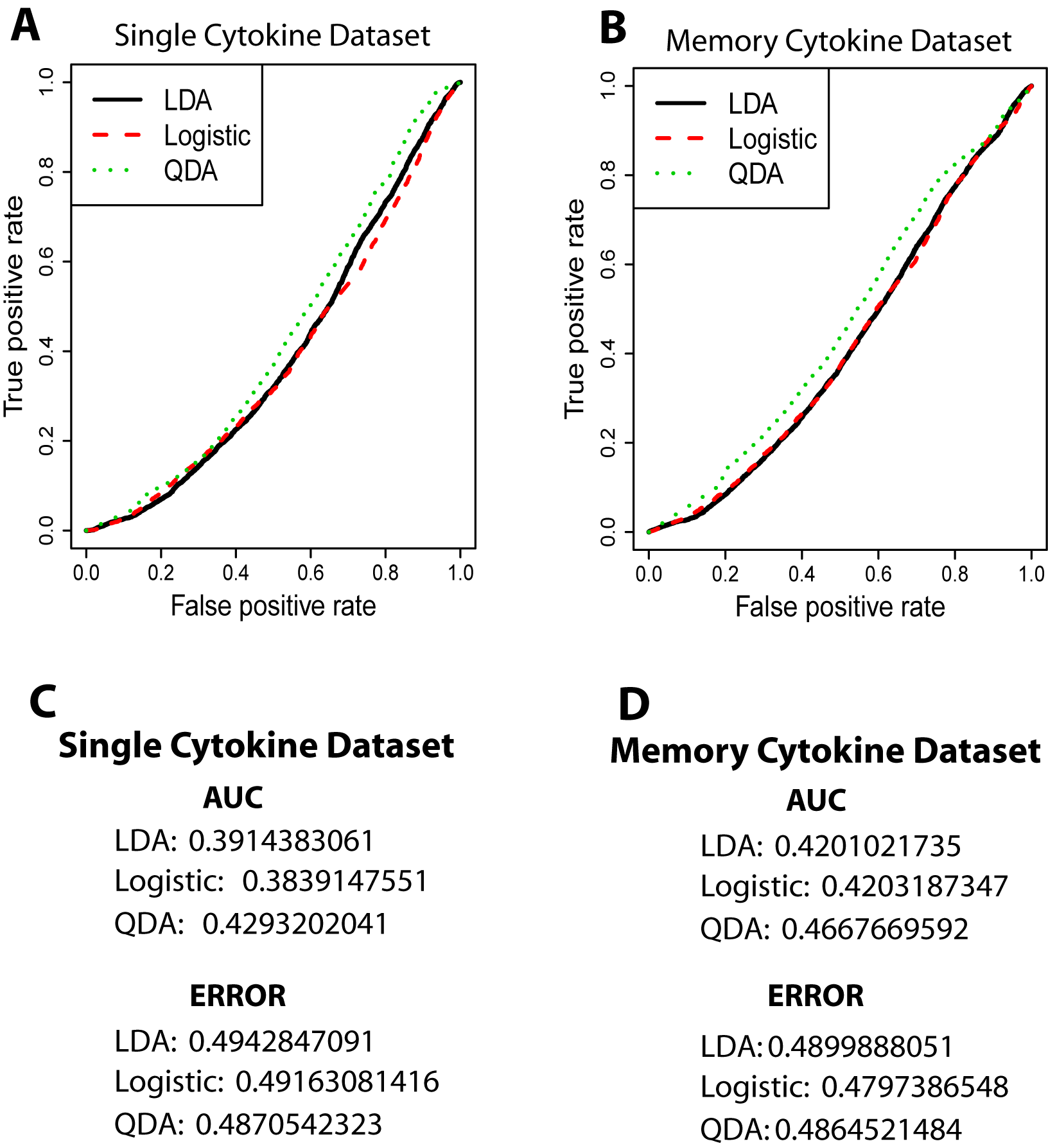

Supplement: S2 Fig — The performances of the binary classification algorithms shown in Table 2 have been measured on the single and memory cytokine datasets by calculating their receiving operating characteristic (ROC) curves (Panels A and B). The area under the curve (AUC) and misclassification error values ere shown in Panels C and D. The script to generate the ROCs have been written in R, using the library “ROCR” and the performance function with true (i.e., tpr) and false positive rates (i.e., fpr) arguments for the cost function (e.g., performance(pred,"tpr","fpr")). The cost associated with tpr and fpr is the same. (TIF) [file pcbi.1004804.s004.tif]

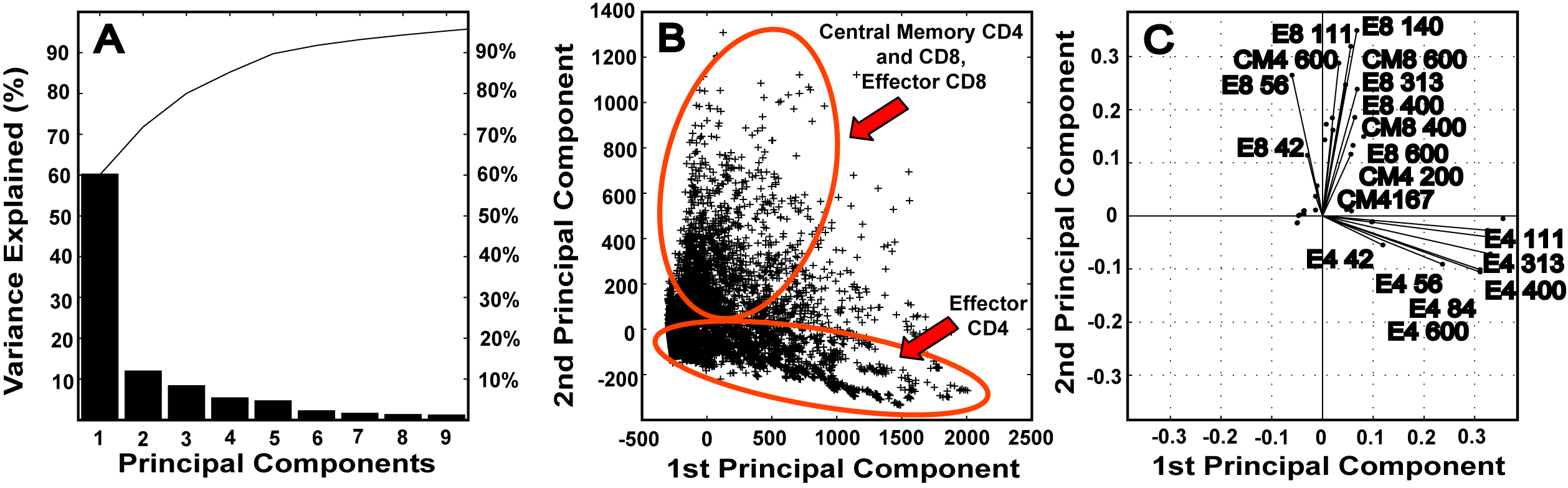

Supplement: S3 Fig — (A-C): Principal Component Analysis (PCA) performed only on Mtb-specific variables in the blood compartment from the same repository of 10,000 in silico granuloma simulations used to generate Fig 4. (A): Pareto plot of the top 10 PCAs. We can reach ~70% of explained variance by summing the top 2 PCAs. Panel B: scatterplot of PCA1 and PCA2. Panel C: biplot associated to Panel B, with categories listed on the four quadrants. Each category is listed as Mtb-specific T cell phenotype at a certain time point post infection. For example “E8 42” refers to Effector CD8+ T cells at day 42 post infection. (Other T cell phenotypes shown: CM [central memory]). (TIF) [file pcbi.1004804.s005.tif]

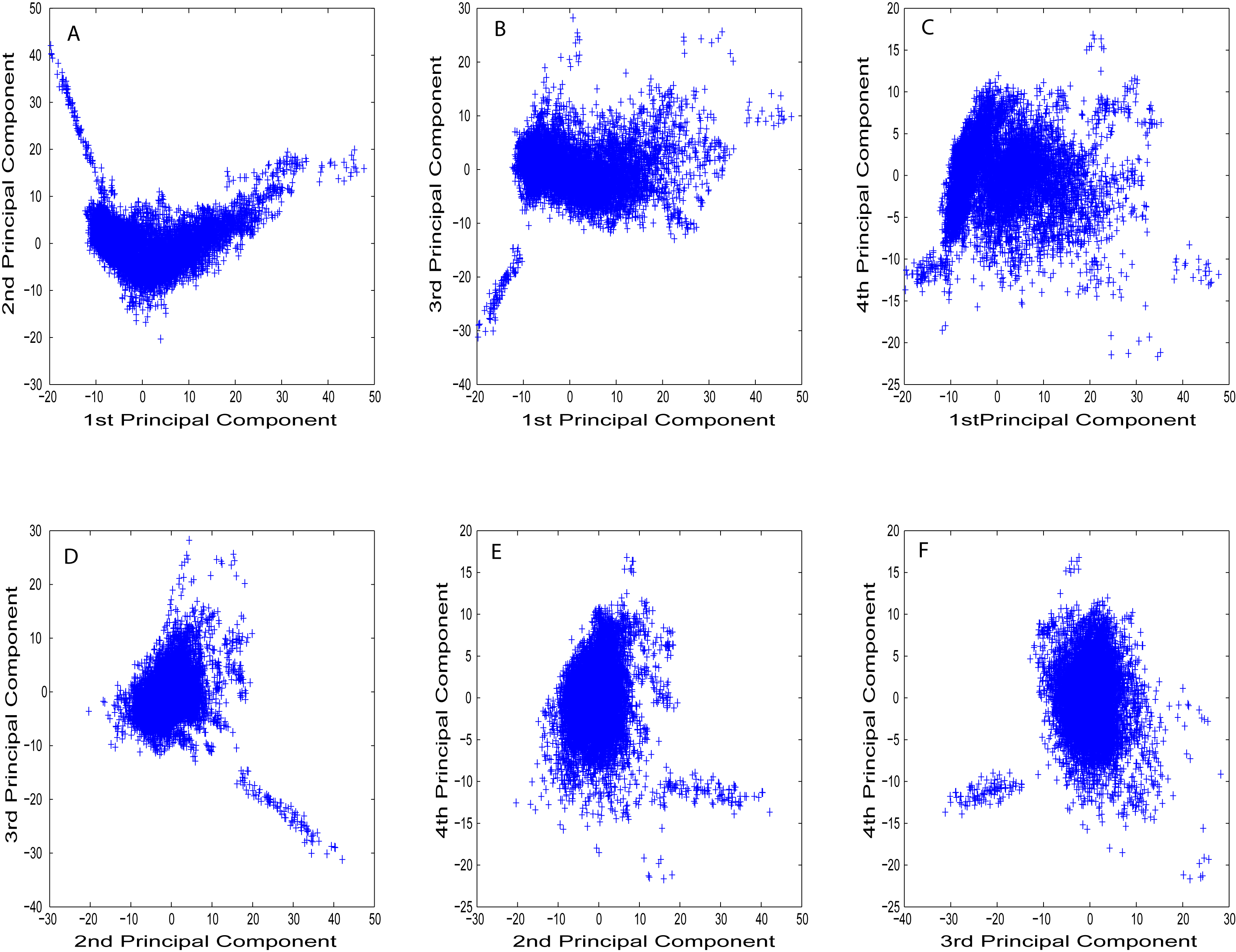

Supplement: S4 Fig — Blood and Lung readouts (49 readouts total). (A)-(C): scatter plots of the 1st principal component versus the 2nd, 3rd and 4th principal component, respectively. (D)-(E): scatter plots of the 2nd principal components versus the 3rd and 4th principal components. (F): scatter plot of the 3rd and 4th principal components. (TIF) [file pcbi.1004804.s006.tif]

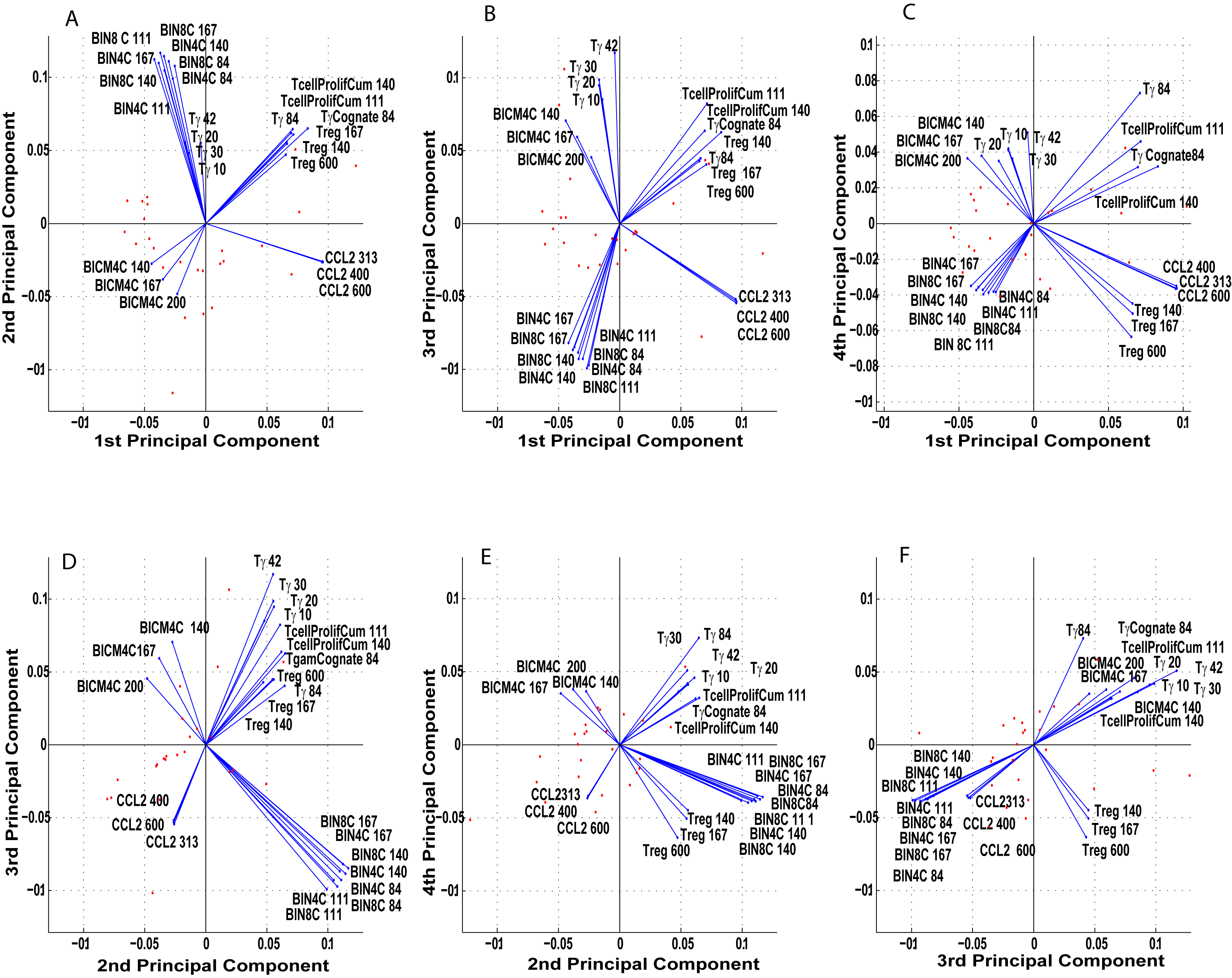

Supplement: S5 Fig — See S11 Table for details on the labels of the scores. The number after the underscore sign refer to the day after infection on which that variable as been measured. We plot the top 4 principal components because they explain ~60 of the variability. (A)-(C): biplots of the scores associated with the scatter plots of the 1st principal component versus the 2nd, 3rd and 4th principal component (as shown in S4 Fig, panels (A)-(C)), respectively. (D)-(E): biplots of the scores associated with the scatter plots of the 2nd principal components versus the 3rd and 4th principal components (as shown in S4 Fig, panels (D)-(E)). (F): biplot of the scores associated with the scatter plot of the 3rd and 4th principal components (as shown in S4 Fig, panel (F)). (TIF) [file pcbi.1004804.s007.tif]

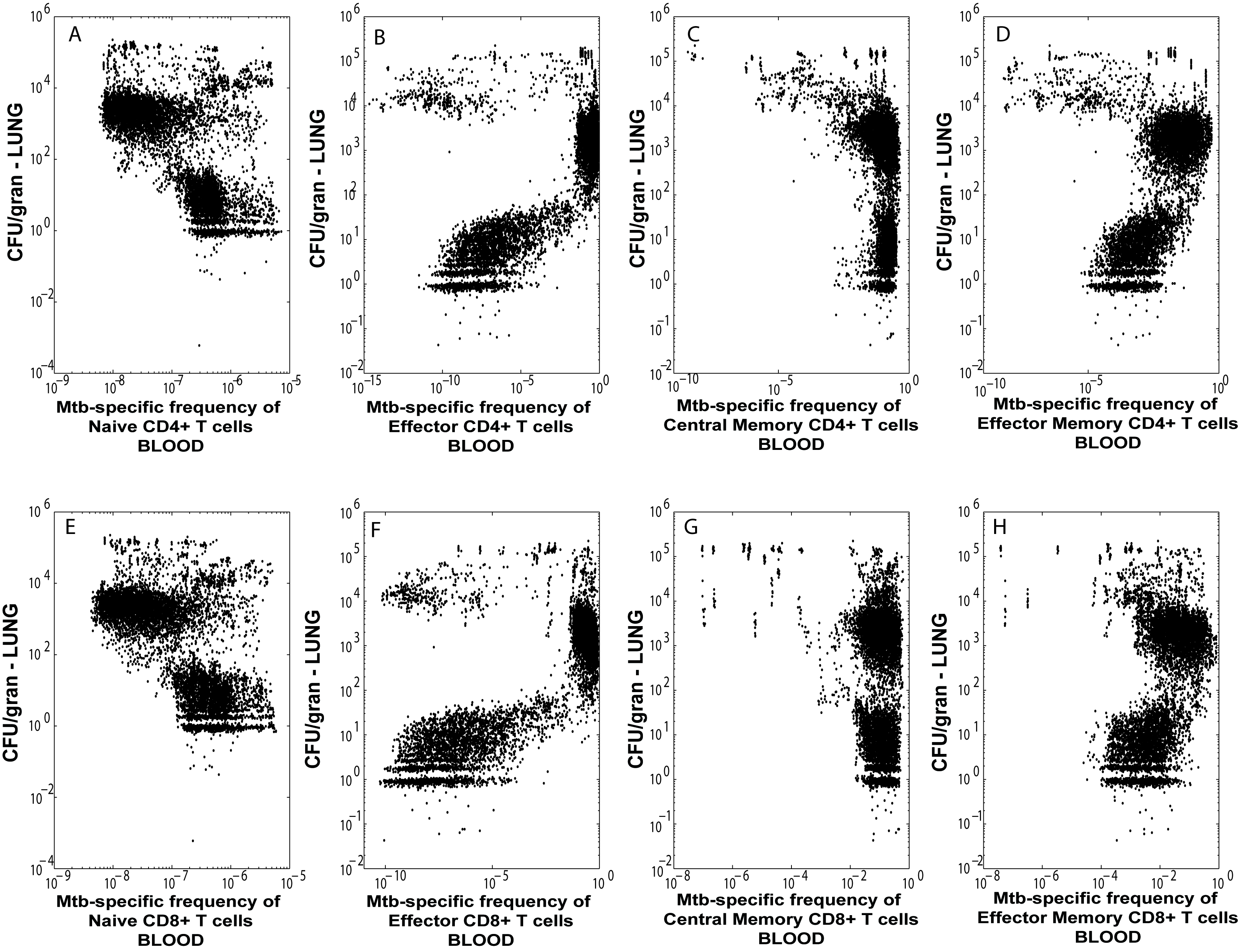

Supplement: S6 Fig — Each panel shows the same repository of 10,000 in silico granuloma simulations coupled to the blood and LN dynamics used to generate Figs 3 and 4. Each point on the plots represents one in silico granuloma. Here we couple information from both the blood (x-axis) and the lung (y-axis). The y-axis represents CFU/granuloma, while the x-axis is the ratio of Mtb-specific vs non Mtb-specific Effector CD4+ cell levels in the blood at day 167 post infection. Both axis are displayed on a log scale. Panels B and F are used in S7 Fig (panels C and D) for detailed studies. (A)-(D): scatter plots of CFU per granuloma (y-axis) versus Mtb-specific frequencies of different CD4+ T cell phenotypes (i.e., Naïve, Effector, Central Memory and Effector Memory). (E)-(H): scatter plots of CFU per granuloma (y-axis) versus Mtb-specific frequencies of different CD8+ T cell phenotypes (i.e., Naïve, Effector, Central Memory and Effector Memory). (TIF) [file pcbi.1004804.s008.tif]

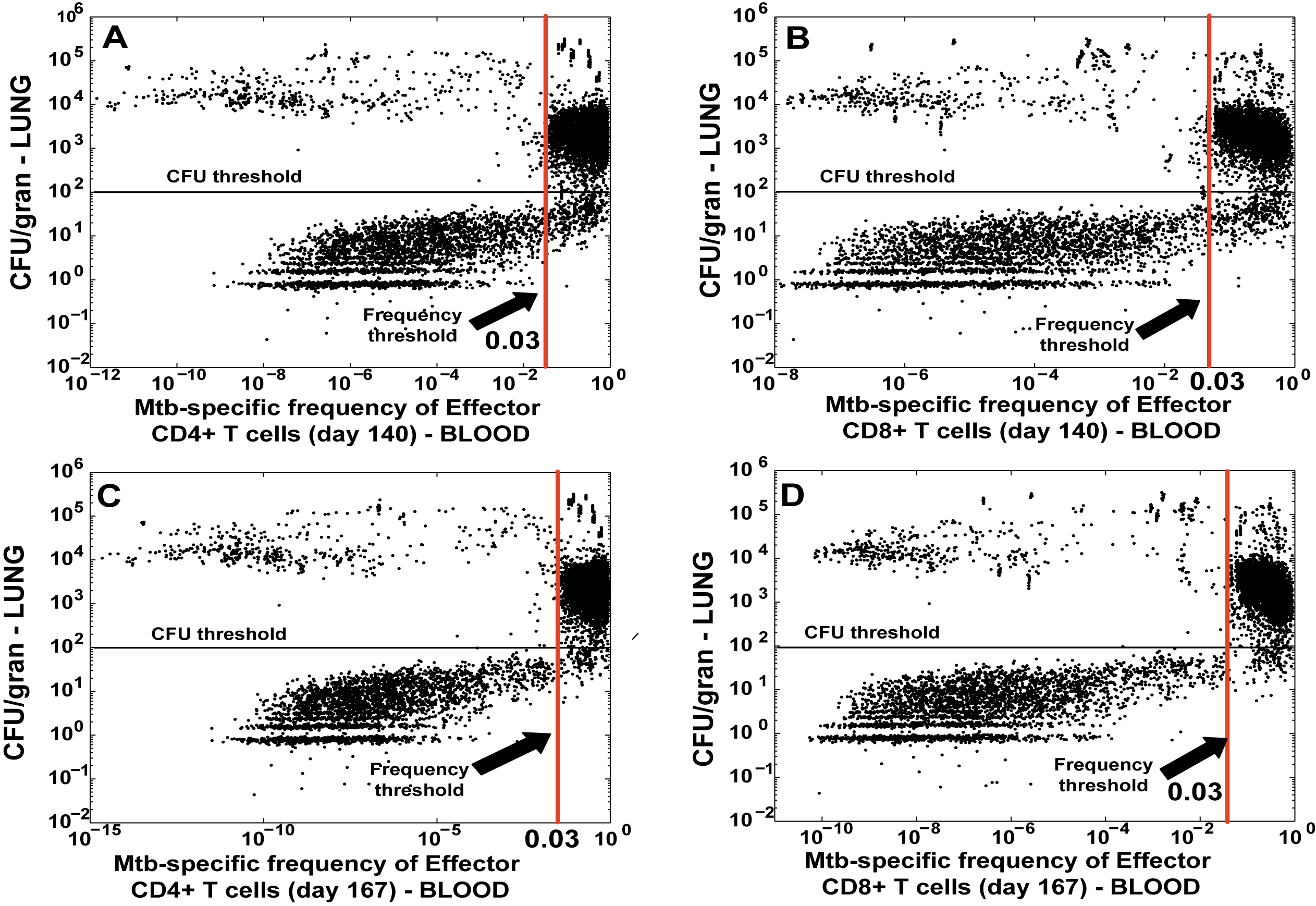

Supplement: S7 Fig — (A-D): Scatter plots of the same repository of 10,000 in silico granuloma simulations coupled to the blood and LN dynamics used to generate Figs 3 and 4. Each point on the plots represents one in silico granuloma. Here we couple information from both the blood (x-axis) and the lung (y-axis). The y-axis represents CFU/granuloma, while the x-axis is the Mtb-specific frequency of Effector CD4+ (A—day 140 / C—day 167) and CD8+ (B—day 140 / D—day 167) cell levels in the blood (A—day 140 / B—day 167). Mtb-specific frequency is calculated by dividing the number of Mtb-specific cells over the total T cells, within each specific phenotype. So, for example the values on the x axis of Panel D are calculated by dividing Mtb-specific Effector CD4+ T cell counts by the total Effector CD4+ T cell counts. Both axis are displayed on a log scale. The horizontal black lines are located at 100 CFU per granuloma and they separate granuloma clusters emerging from the 10,000 in silico simulations as either low or high CFU granulomas. Vertical red lines are the Mtb-specific frequency thresholds suggested in each panel to predict granuloma with low versus high CFU. We chose these two time points (i.e., day 140 and day 167) as they displayed the best separation between granuloma clusters with low versus high CFU burden. All other Mtb-specific frequencies that can be calculated on the in silico data (namely, naïve, effector, central and effector memory ratios for CD4+ and CD8+ T cells) and plotted against CFU/granuloma at the latest time point measured in the memory dataset (i.e., day 167 post infection) are shown in S6 Fig. There is a small number of granulomas that do not fall into either these groups, (i.e., where high CFU is associated with low Mtb-specific T cell frequencies, false positive group if we use the frequency threshold as a biomarker). (TIF) [file pcbi.1004804.s009.tif]

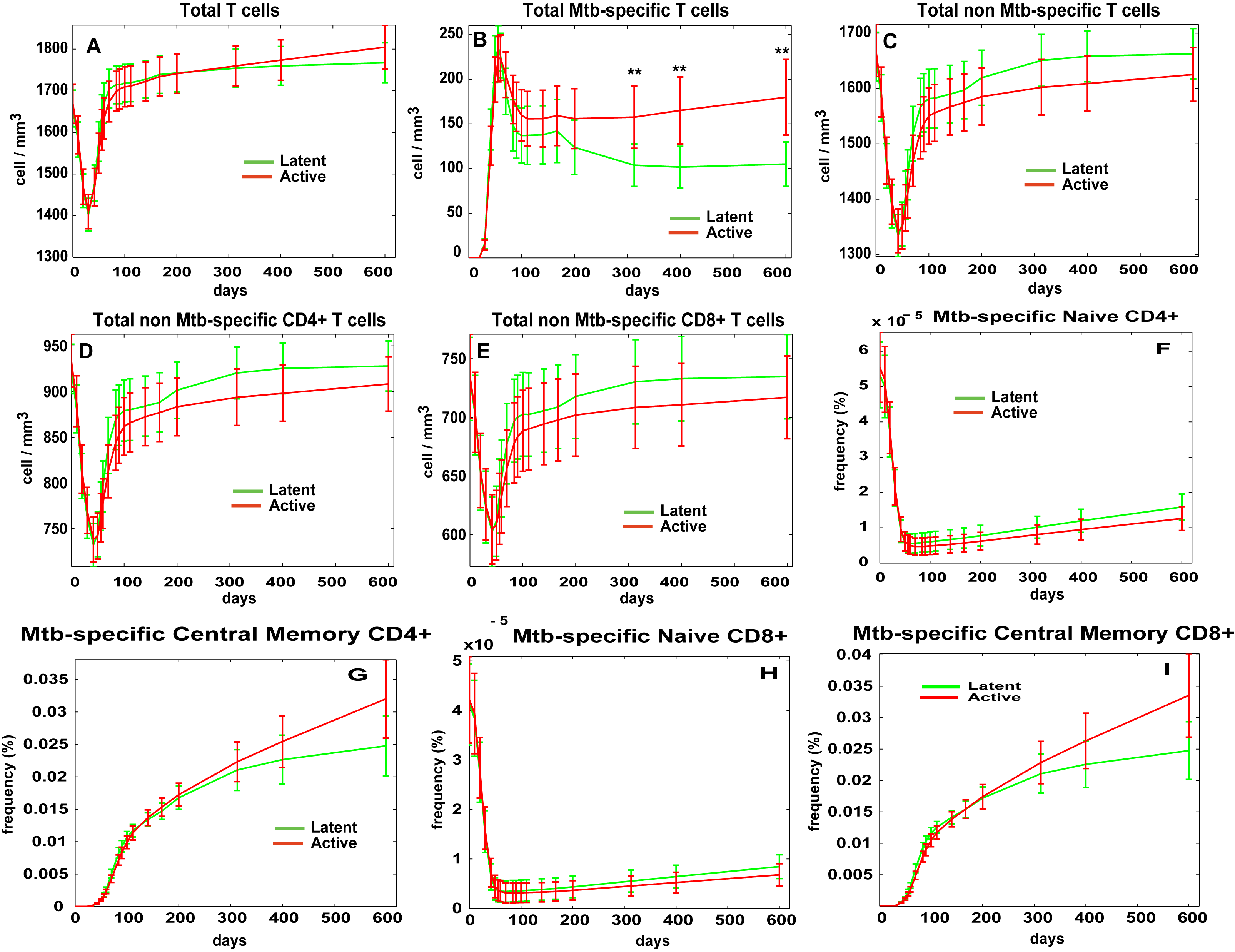

Supplement: S8 Fig — Trajectories over 600 days of T cell levels, both total and Mtb-specific, as well as Mtb-specific T cell frequencies. The data shown have been generated following steps illustrated in Fig 6, as well as in the Materials and Methods section. The 43 virtual NHPs have been classified based on known clinical outcome and are displayed in all the panels as mean +/- 2x(standard error). The asterisks show significant (p<0.05) student t-test between the two trajectories at the same time point. Panel A: Total T cell levels. Panel B: Total CD4+ T cell levels. Panel B: Total Mtb-specific T cell levels. Panel C: Total non Mtb-specific T cell levels. Panel D: Total non Mtb-specific CD4+ T cell levels. Panel E: Total non Mtb-specific CD8+ T cell levels. Panel F: Frequency of Mtb-specific Naïve CD4+ T cells. Panel G: Frequency of Mtb-specific Central Memory CD4+ T cells. Panel H: Frequency of Mtb-specific Naïve CD8+ T cells. Panel I: Frequency of Mtb-specific Central Memory CD8+ T cells. (TIF) [file pcbi.1004804.s010.tif]

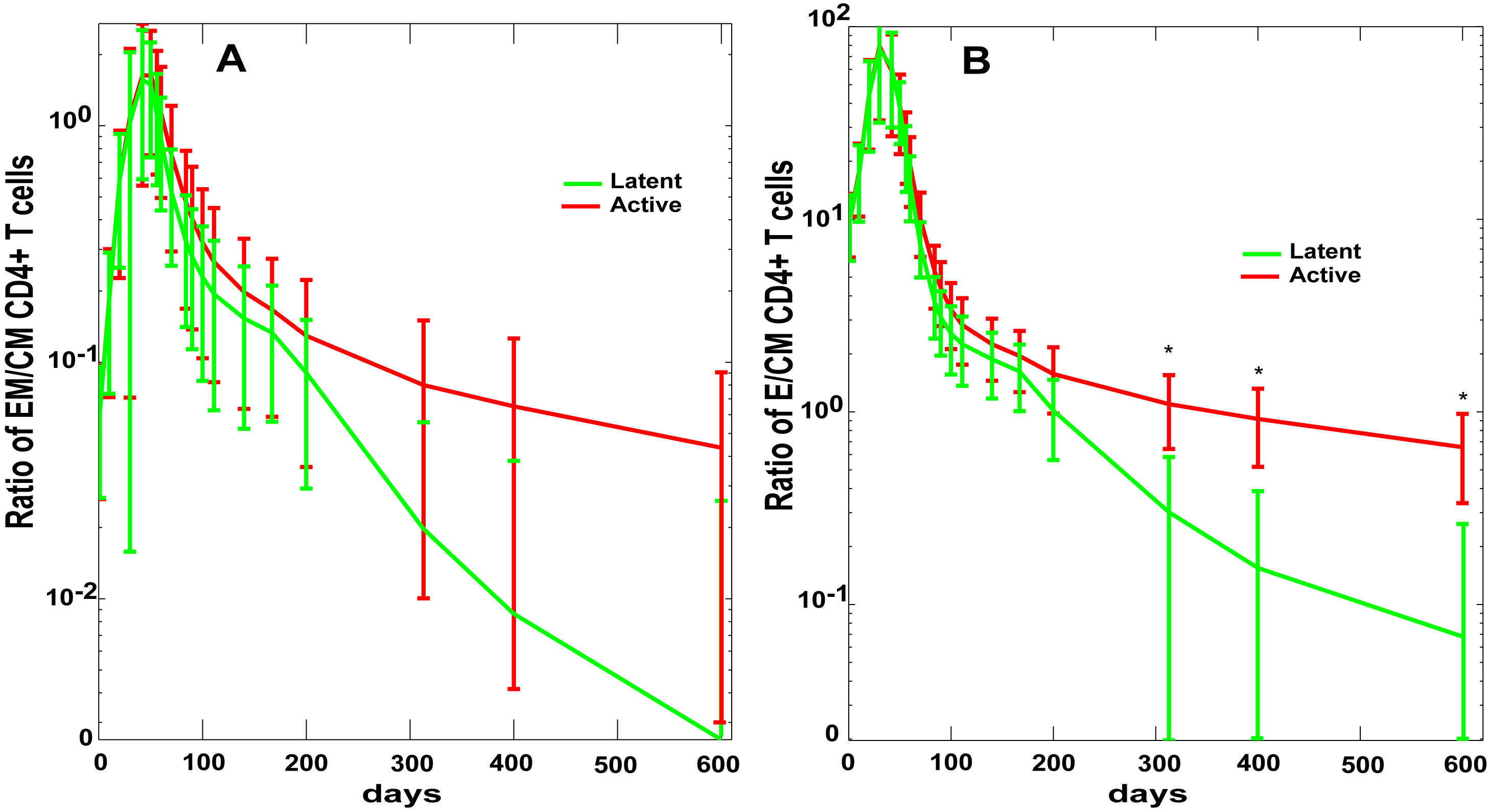

Supplement: S9 Fig — Trajectories over 600 days of ratios of Mtb-specific CD4+ T cell levels. The data shown have been generated following steps illustrated in of 6, as well as in the Materials and Methods section. The 43 virtual NHPs have been classified based on known clinical outcome and are displayed in all the panels as mean +/- 2x(standard error). The asterisks show significant (p<0.05) student t-test between the two trajectories at the same time point. Panel A: Ratio of Effector Memory over Central Memory Mtb-specific CD4+ T cells. Panel B: Ratio of Effector over Central Memory Mtb-specific CD4+ T cells. (TIF) [file pcbi.1004804.s011.tif]
