# Supplementary material for: Computational and Empirical Studies Predict Mycobacterium tuberculosis-Specific T Cells as a Biomarker for Infection Outcome
Source: PLoS Comput Biol. 2016 Apr 11;12(4):e1004804. doi: 10.1371/journal.pcbi.1004804 (PMC4827839; doi:10.1371/journal.pcbi.1004804)
Supplement: S7 Table — (PDF) [file pcbi.1004804.s018.pdf]

NHP ID	Clinical classification	k-means	Hclust-Ward	Hclust-Complete	HClust-Avg	MDS
1512	Active	1	1	1	1	1
1712	Active	1	2	2	2	2
1812	Active	1	1	1	1	1
2112	Latent	2	3	3	3	3
2312	Latent	1	1	4	1	1
2612	Latent	1	1	5	4	1
2712	Latent	1	1	1	1	1
17211	Latent	1	1	4	1	1
17411	Active	1	3	3	3	4
21310	Active	3	2	6	5	5
21410	Active	4	2	6	5	5
21510	Latent	5	2	7	6	6
21610	Latent	5	2	7	6	6
21710	Latent	1	2	6	5	6
21810	Active	2	3	3	3	3
21910	Latent	2	3	3	3	3
22010	Active	6	2	8	7	7
22210	Latent	7	3	3	3	3
22310	Active	1	2	9	8	4
22410	Latent	1	2	10	9	7
22510	Active	1	2	11	10	7
22610	Latent	1	2	8	7	1
22710	Active	1	2	12	11	2
22810	Latent	8	2	13	12	2
22910	Active	9	2	9	8	7
23010	Latent	1	2	14	13	7
23110	Active	1	2	11	14	5
23210	Active	1	1	1	15	1
**Optimal # of clusters**		9	3	14	15	7

Table 5: **results for the unsupervised classification algorithms applied to the *single cytokine dataset*** Similar multiple optimal clusters have been returned by applying the five unsupervised algorithms to the multiple cytokine and memory phenotype temporal datasets (data not shown).
