# Supplementary material for: Computational and Empirical Studies Predict Mycobacterium tuberculosis-Specific T Cells as a Biomarker for Infection Outcome
Source: PLoS Comput Biol. 2016 Apr 11;12(4):e1004804. doi: 10.1371/journal.pcbi.1004804 (PMC4827839; doi:10.1371/journal.pcbi.1004804)
Supplement: S8 Table — (PDF) [file pcbi.1004804.s019.pdf]

Variable	Value	Units	Description
APC	0	Cell count	Antigen presenting cell proxy in the lymph node
$N_{Ln,4}$	$N_{B,4}$ x (α/ host_Ln)	Cell count	Lymph node naïve CD4+
$P_{Ln,4}$	0	Cell count	Lymph node precursor CD4+
$EM_{Ln,4}$	0	Cell count	Lymph node effector memory CD4+
$CM_{Ln,4}$	0	Cell count	Lymph node central memory CD4+
$N_{B,4}$	[365,1213] x λ	Cell/mm^3	Blood naïve CD4+
$E_{B,4}$	0	Cell/mm^3	Blood effector CD4+
$CM_{B,4}$	0	Cell/mm^3	Blood central memory CD4+
$EM_{B,4}$	0	Cell/mm^3	Blood effector memory CD4+
$N_{Ln,8}$	$N_{B,8}$ x (α/ host_Ln)	Cell count	Lymph node naïve CD8+
$P_{Ln,8}$	0	Cell count	Lymph node precursor CD8+
$EM_{Ln,8}$	0	Cell count	Lymph node effector memory CD8+
$CM_{Ln,8}$	0	Cell count	Lymph node central memory CD8+
$N_{B,8}$	[117,825] x λ	Cell/mm^3	Blood naïve CD8+
$E_{B,8}$	0	Cell/mm^3	Blood effector CD8+
$CM_{B,8}$	0	Cell/mm^3	Blood central memory CD8+
$EM_{B,8}$	0	Cell/mm^3	Blood effector memory CD8+
$N_{Ln,nc4}$	$N_{B,nc4}$ x (α/ host_Ln)	Cell count	Non-cognate lymph node naïve CD4+
$CM_{Ln,nc4}$	$CM_{B,nc4}$ x (α/ host_Ln)	Cell count	Non-cognate lymph node central memory CD4+
$N_{B,nc4}$	[365,1213] x (1-λ)	Cell/mm^3	Non-cognate blood naïve CD4+
$E_{B,nc4}$	[70,662]	Cell/mm^3	Non-cognate blood effector CD4+
$CM_{B,nc4}$	[136,447]	Cell/mm^3	Non-cognate blood central memory CD4+
$EM_{B,nc4}$	[51,325]	Cell/mm^3	Non-cognate blood effector memory CD4+
$N_{Ln,nc8}$	$N_{B,nc8}$ x (α/ host_Ln)	Cell count	Non-cognate lymph node naïve CD8+
$CM_{Ln,nc8}$	$CM_{B,nc8}$ x (α/ host_Ln)	Cell count	Non-cognate lymph node central memory CD8+
$N_{B,nc8}$	[117,825]	Cell/mm^3	Non-cognate blood naïve CD8+
$E_{B,nc8}$	[50,486]	Cell/mm^3	Non-cognate blood effector CD8+
$CM_{B,nc8}$	[52,658]	Cell/mm^3	Non-cognate blood central memory CD8+
$EM_{B,nc8}$	11,179]	Cell/mm^3	Non-cognate blood effector memory CD8+

Table 2. The initial conditions used in the blood and lymph node ODE compartments. 1 mm^3 = 0.001 ml = 1 μl
