# Supplementary material for: Computational and Empirical Studies Predict Mycobacterium tuberculosis-Specific T Cells as a Biomarker for Infection Outcome
Source: PLoS Comput Biol. 2016 Apr 11;12(4):e1004804. doi: 10.1371/journal.pcbi.1004804 (PMC4827839; doi:10.1371/journal.pcbi.1004804)
Supplement: S9 Table — (PDF) [file pcbi.1004804.s020.pdf]

Parameter	Value	Units	Description
α	5.6e5	μL	Conversion factor from Blood to Ln (max. blood volume)
host_Ln	50	count	Number of lymph nodes in the host
λ	[1e-5 , 1e-3]	“”	Mtb-specific frequency
Sn4*	$N_{Ln,4}$ x (α/ host_Ln)	Cell/μL*day	Thymic output of naive CD4 cells
Sn8*	$N_{Ln,8}$ x (α/ host_Ln)	Cell/μL*day	Thymic output of naive CD8 cells
hs_1	25	Cell count	Naive CD4 recruitment half saturation
hs_4	10	Cell count	Precursor CD4 proliferation half saturation
hs_5	10	Cell count	Precursor CD4 differentiationhalf saturation
hs_8	40	Cell count	Central Memory CD4 recruitment half saturation
hs_{10}	357.7	Cell count	Naive CD8 recruitment half saturation
hs_{11}	10	Cell count	Naive CD8 priming half saturation
hs_{13}	10	Cell count	Precursor CD8 proliferation half saturation
hs_{14}	10	Cell count	Precursor CD8 differentiation half saturation
hs_{17}	157	Cell count	Central Memory CD8 recruitment half saturation
k_1	[5e-1,1]	day^{-1}	Naive CD4 recruitment rate
k_2	[1e-3,1e-1]	day^{-1}	Naive CD4 Priming rate
k_3	[1e-7,1e-4]	day^{-1}	Central Memory CD4 reactivation rate
k_4	[.5,1.5]	day^{-1}	Precursor CD4 proliferation rate
k_5	[0.01,0.5]	day^{-1}	Precursor CD4 differentiation to Effector rate
k_6	0.004	day^{-1}	Precursor CD4 differentiation to Central Memory
k_7	[0.005,0.1]	day^{-1}	Effector CD4 differentiation to Effector Memory
k_8	[0.1,0.31]	day^{-1}	Central Memory CD4 recruitment rate
k_{10}	[5e-1,1]	day^{-1}	Naive CD8 recruitment rate
k_{11}	[1e-3,1e-1]	day^{-1}	Naive CD8 priming rate
k_{12}	[1e-7,1e-4]	day^{-1}	Central Memory CD8 reactivation rate
k_{13}	[.5,1.5]	day^{-1}	Precursor CD8 proliferation rate
k_{14}	[0.01,0.5]	day^{-1}	Precursor CD8 differentiation to Effector rate
k_{15}	0.004	day^{-1}	Precursor CD8 differentiation to Central Memory
k_{16}	[0.005,0.1]	day^{-1}	Effector CD8 differentiation to Effector Memory
k_{17}	0.5	day^{-1}	Central Memory CD8 recruitment rate
μ_1	0.2	day^{-1}	Effector CD4 death rate
μ_2	0.04	day^{-1}	Effector Memory CD4 death rate
μ_3	0.2	day^{-1}	Effector CD8 death rate
μ_4	0.04	day^{-1}	Effector Memory CD8 death rate
μ_5	[0.1,1]	day^{-1}	APC death rate
μ_6	0.1	day^{-1}	Precursor CD4 death rate
μ_7	0.1	day^{-1}	Precursor CD8 death rate
μ_8	6e-4	day^{-1}	Naïve CD4 death rate
μ_9	4e-4	day^{-1}	Naïve CD8 death rate
ρ_1	3e8	Cell count	Precursor carrying capacity
Wp_4	0.735	“”	Weight factor for Precursor CD4 in CD8 priming
ξ_1*	ξ_2 x ($N_{Ln,nc4}$/ $N_{B,nc4}$) / α	day^{-1}	Naïve CD4 Lymph Influx
ξ_2	[0.637,1]	day^{-1}	Naive CD4 Lymph Efflux
ξ_3	[2,5]	day^{-1}	Effector CD4 Lymph Efflux
ξ_4*	ξ_5 x ($CM_{Ln,nc4}$/ $CM_{B,nc4}$) / α	day^{-1}	Central Memory CD4 Lymph Influx
ξ_5	0.489	day^{-1}	Central Memory CD4 Lymph Efflux
ξ_6	[2,5]	day^{-1}	Effector Memory CD4 Lymph Efflux
ξ_7*	ξ_8x ($N_{Ln,nc8}$/ $N_{B,nc8}$) / α	day^{-1}	Naïve CD8 Lymph Influx
ξ_8	[0.643,1]	day^{-1}	NaiveCD8 Lymph Efflux
ξ_9	[2,5]	day^{-1}	Effector CD8 Lymph Efflux
ξ_{10}*	ξ_{11} x ($CM_{Ln,nc8}$/ $CM_{B,nc8}$)/ α	day^{-1}	Effector CD8 Lymph Influx
ξ_{11}	[2,5]	day^{-1}	Central Memory CD8 Lymph Efflux
ξ_{12}	[2,5]	day^{-1}	Effector Memory CD8 Lymph Efflux
proliferationTime	8	hrs	Doubling time for cognate T cells in the lung
maxDivisions	4	--	Max number of divisions for T cells in the lung

Supplementary Table 9. The baseline parameters used in the blood, lymph node ODE compartments. *Parameters calculated based upon the initial conditions of the system and the corresponding lymph efflux term. These parameters were not varied during LHS experiments as they were constrained by a corresponding lymph efflux parameter and the assumption that our initial conditions meet homeostasis.
