# Supplementary material for: Computational and Empirical Studies Predict Mycobacterium tuberculosis-Specific T Cells as a Biomarker for Infection Outcome
Source: PLoS Comput Biol. 2016 Apr 11;12(4):e1004804. doi: 10.1371/journal.pcbi.1004804 (PMC4827839; doi:10.1371/journal.pcbi.1004804)
Supplement: S10 Table — (DOC) [file pcbi.1004804.s021.doc]

**Supplementary Table 10 text**

Supplementary Figure 6 shows scatter plots of Mtb-specific frequencies of in silico CD4+ and CD8+ Effector T cells with the corresponding combined *in silico* granuloma CFU burdens from our repository of 10,000 granulomas coupled to blood and LN dynamics. Two clusters of granulomas emerge, separated by a CFU/granuloma threshold (black horizontal lines in Supplementary Fig. 7A-D). Granulomas that can control bacterial growth are defined as *low* if CFU/granuloma<100 at any time after day 140 post infection, and *high* otherwise. It is possible to separate the low and high CFU groups by tracking Mtb-specific T cell frequencies (i.e., the number of Mtb-specific T cells over total T cells, for each phenotype) on the x-axis. If Mtb-specific effector CD4+ T cell frequency in blood at day 140 or later is below 3% (or 0.03, vertical red lines in Fig. 4A and 4C, labeled as Mtb-specific *frequency threshold*), we predict that an *in silico* granuloma will progress to a low CFU and/or sterilize with probability ~90-91% (Supplementary Table 9 below for details). Mtb-specific effector CD8+ T cell frequencies in the blood have a similar performance in predicting low combined CFU/granuloma outcomes (Fig. 4B and 4D, and Supplementary Table 9 below for details). The accuracy improves if we combine the Mtb-specific CD4+ and CD8+ effector T cell frequencies (i.e, below 3%, see Supplementary Table 9 below for details). Overall, our approach identifies two blood biomarkers to predict granuloma-scale outcomes: i) Mtb-specific effector CD4+ T cell frequency and ii) Mtb-specific effector CD8+ T cell frequency.

|  | **E4 ratio** | | **E8 ratio** | | **E4 + E8 ratios** | |
| --- | --- | --- | --- | --- | --- | --- |
|  | ***CFU/granuloma*** | | ***CFU/granuloma*** | | ***CFU/granuloma*** | |
|  | ***LOW*** | ***HIGH*** | ***LOW*** | ***HIGH*** | ***LOW*** | ***HIGH*** |
| ***Day 84*** | ~73.5% | ~55.2% | ~74.5% | ~52.4% | ~85.5% | ~55.8% |
| ***Day 111*** | ~86.6% | ~77.8% | ~87% | ~74.4% | ~91.2% | ~79.3% |
| ***Day 140*** | ~90.5% | ~92% | ~90.3% | ~91.6% | ~92.8% | ~92.3% |
| ***Day 167*** | ~91.1% | ~97% | ~90.1% | ~97% | ~93% | ~97% |
| ***Day 200*** | ~91.2% | ~99% | ~90.7% | ~99% | ~92.6% | ~99.1% |
| ***Day 313*** | ~89.3% | ~99.8% | ~89.3% | ~99.85% | ~90.1% | ~99.85% |
| ***Day 446*** | ~86.3% | ~99.88% | ~86.2% | ~99.87% | ~86.8% | ~99.87% |
| ***Day 481*** | ~85.4% | ~99.87% | ~85.4% | ~99.87% | ~86% | ~99.88% |

**Supplementary Table 10:** In silico biomarkers accuracy in predicting CFU/granuloma. Low (High) CFU classification is based on Total CFU per granuloma < (>) 100 Mtb. Total CFU per granuloma is established at day 200 post infection. No significant differences emerge if the classification is based on Total CFU at later days post infection. The thresholds for the Mtb-specific frequencies of Effector CD4+ and CD8+ T cells is 3% or 0.03. For example, in the first column at day 111, if the frequency of Mtb-specific Effector CD4+ T cells is less than 3%, the in silico granuloma will become a low CFU one with a likelihood of 86.6% and if it is greater than 3%, the likelihood of being a high CFU granuloma is 77.8%.
