# Supplementary material for: Computational and Empirical Studies Predict Mycobacterium tuberculosis-Specific T Cells as a Biomarker for Infection Outcome
Source: PLoS Comput Biol. 2016 Apr 11;12(4):e1004804. doi: 10.1371/journal.pcbi.1004804 (PMC4827839; doi:10.1371/journal.pcbi.1004804)
Supplement: S11 Table — (PDF) [file pcbi.1004804.s022.pdf]

Supplementary Table 10: 49 readouts of the computational model used for PCA.

Computational Model Variable	Description	Computational Model Variable	Description
Mac	Total number of macrophages in the lung compartment	*LnCM8C*	Eqn 1.14: Mtb-specific CD8+ Central Memory in the lymph node
Tγ	Total number of Ty cells in the lung compartment	*LnEM8C*	Eqn 1.15: Mtb-specific CD8+ Effector Memory in the lymph node
Tcyt	Total number of Cytotoxic T cells in the lung compartment	*BlN8C*	Eqn 1.16: Mtb-specific CD8+ in the blood
Treg	Total number of Regulatory T cells in the lung compartment	*BlE8C*	Eqn 1.17: Mtb-specific CD8+ Effector in the blood
TγCognate	Total number of Mtb-specific Ty cells in the lung compartment	*BlCM8C*	Eqn 1.18: Mtb-specific CD8+ Central Memory in the blood
TcytCognate	Total number of Mtb-specific Cytotoxic T cells in the lung compartment	*BlEM8C*	Eqn 1.19: Mtb-specific CD8+ Effector Memory in the blood
TotMtb.	Total CFU/granuloma	*LnN4NC*	Eqn 1.20: non Mtb-specific CD4+ in the lymph node
TNF	TNF concentration in the Lung compartment	*LnCM4NC*	Eqn 1.21: non Mtb-specific CD4+ Central Memory in the lymph node
IL10	IL10 concentration in the Lung compartment	*BlN4NC*	Eqn 1.22: non Mtb-specific CD4+ Naïve cells in the blood
CCL2	CCL2 concentration in the Lung compartment	*BlE4NC*	Eqn 1.23: non Mtb-specific CD4+ in the blood
LesionSize	Lesion size of the granuloma (mm)	*BlCM4NC*	Eqn 1.24: non Mtb-specific CD4+ Central Memory in the blood
APC	Eqn 1.1 – Antigen Presenting Cell proxy in the lymph node	*BlEM4NC*	Eqn 1.25: non Mtb-specific CD4+ Effector Memory in the blood
LnN4C	Eqn 1.2: Mtb-specific CD4+ Naïve cells in the lymph node	*LnN8NC*	Eqn 1.26: non Mtb-specific CD8+ in the lymph node
LnP4C	Eqn 1.3: Mtb-specific CD4+ in the lymph node	*LnCM8NC*	Eqn 1.27: non Mtb-specific CD8+ Central Memory in the lymph node
LnE4C	Eqn 1.4: Mtb-specific CD4+ Effector in the lymph node	*BlN8NC*	Eqn 1.28: non Mtb-specific CD8+ Naïve cells in the blood
LnCM4C	Eqn 1.5: Mtb-specific CD4+ Central Memory in the lymph node	*BlE8NC*	Eqn 1.29: non Mtb-specific CD8+ Effector in the blood
LnEM4C	Eqn 1.6: Mtb-specific CD4+ Effector Memory in the lymph node	*BlCM8NC*	Eqn 1.30: non Mtb-specific CD8+ Central Memory in the blood
BlN4C	Eqn 1.7: Mtb-specific CD4+ Naïve cells in the blood	*BlEM8NC*	Eqn 1.31: non Mtb-specific CD8+ Effector Memory in the blood
BlE4C	Eqn 1.8: Mtb-specific CD4+ Effector in the blood	*TγRecruited*	Cumulative number of Tg cells recruited in the Lung compartment
BlCM4C	Eqn 1.9: Mtb-specific CD4+ Central Memory in the blood	*TcytRecruited*	Cumulative number of Cytotoxic T cells recruited in the Lung compartment in the Lung compartment
BlEM4C	Eqn 1.10: Mtb-specific CD4+ Effector Memory in the blood	*Fas_FasL_Kill*	Cumulative number of macrophage killing due to FAS/FASL apoptosis in the Lung compartment
LnN8C	Eqn 1.11: Mtb-specific CD8+ Naïve cells in the lymph node	*CTL_Killing*	Cumulative number of macrophage killing due to Cytotoxic T cells in the Lung compartment
LnP8C	Eqn 1.12: Mtb-specific CD8+ in the lymph node	*Mci_Burst*	Cumulative number of chronically infected macrophage bursting in the Lung compartment
LnE8C	Eqn 1.13: Mtb-specific CD8+ in the lymph node	*TcellProlifCum*	Cumulative number of T cell that have been proliferated in the Lung compartment (only Mtb-specific T cells can proliferate)
		CFU	Total CFU per granuloma
